# Dietary protein-induced hepatic IGF-1 secretion mediated by PPARγ activation

**DOI:** 10.1371/journal.pone.0173174

**Published:** 2017-03-03

**Authors:** Xiaojuan Wan, Songbo Wang, Jingren Xu, Lu Zhuang, Kongping Xing, Mengyuan Zhang, Xiaotong Zhu, Lina Wang, Ping Gao, Qianyun Xi, Jiajie Sun, Yongliang Zhang, Tiejun Li, Gang Shu, Qingyan Jiang

**Affiliations:** 1 College of Animal Science and National Engineering Research Center for Breeding Swine Industry, South China Agricultural University, Guangzhou, China; 2 Key Laboratory of Subtropical Agro-ecology, Institute of Subtropical Agriculture, The Chinese Academy of Sciences, Changsha, Hunan, PR China; INRA, FRANCE

## Abstract

Dietary protein or amino acid (AA) is a crucial nutritional factor to regulate hepatic insulin-like growth factor-1 (IGF-1) expression and secretion. However, the underlying intracellular mechanism by which dietary protein or AA induces IGF-1 expression remains unknown. We compared the IGF-1 gene expression and plasma IGF-1 level of pigs fed with normal crude protein (CP, 20%) and low-protein levels (LP, 14%). RNA sequencing (RNA-seq) was performed to detect transcript expression in the liver in response to dietary protein. The results showed that serum concentrations and mRNA levels of IGF-1 in the liver were higher in the CP group than in the LP group. RNA-seq analysis identified a total of 1319 differentially expressed transcripts (667 upregulated and 652 downregulated), among which the terms “oxidative phosphorylation”, “ribosome”, “gap junction”, “PPAR signaling pathway”, and “focal adhesion” were enriched. In addition, the porcine primary hepatocyte and HepG2 cell models also demonstrated that the mRNA and protein levels of IGF-1 and PPARγ increased with the increasing AA concentration in the culture. The PPARγ activator troglitazone increased IGF-1 gene expression and secretion in a dose dependent manner. Furthermore, inhibition of PPARγ effectively reversed the effects of the high AA concentration on the mRNA expression of IGF-1 and IGFBP-1 in HepG2 cells. Moreover, the protein levels of IGF-1 and PPARγ, as well as the phosphorylation of mTOR, significantly increased in HepG2 cells under high AA concentrations. mTOR phosphorylation can be decreased by the mTOR antagonist, rapamycin. The immunoprecipitation results also showed that high AA concentrations significantly increased the interaction of mTOR and PPARγ. In summary, PPARγ plays an important role in the regulation of IGF-1 secretion and gene expression in response to dietary protein.

## Introduction

Insulin-like growth factor 1 (IGF-1) is a major regulator of growth and metabolism for mammals; this protein is synthesized and released from the liver [1, 2]. IGF-1 is considered a somatomedin that mediates the effects of growth hormone (GH) and promotes animal growth (Wu *et al*. 2009; Zhang *et al*. 2010; Rotwein 2012; Durzynska *et al*. 2013). Circulating IGF-1 is modulated by IGF binding proteins family (IGFBP-1-6) and acid-labile subunit (ALS) [3, 4], whereas the synthesis and secretion of IGF-1 are dependent on some hormones, such as GH, insulin [5–8], and nutritional status [9].

Several reports have demonstrated that plasma IGF-1 levels decrease in response to protein-calorie malnutrition [9, 10]. Low crude protein (LP) diets (13.6%) decreased the piglet plasma IGF-1 concentrations by approximately 10%, whereas the concentrations of glucose, triglycerides, T3, T4, or GH [11] remained unchanged. By contrast, the plasma IGF-1 concentration increases with the increasing dietary protein content up to 20.7% in growing pigs. The plasma IGF-1 concentration positively correlated with protein accretion to dietary protein content [12]. Additionally, lower protein intake further increased the concentration of IGFBP-1, which is a key binding protein that inhibits IGF-1 activity [10].

The nutritional basis of proteins is amino acids (AAs). A low-protein diet significantly decreased the plasma AA in pigs. A recent study showed that the serum concentration of some essential AAs (e.g., arginine and phenylalanine) and nonessential AAs (e.g., glutamine and cysteine) in pigs fed with a low-protein diet were only 25%–50% of normal levels [13]. The reduced hepatic IGF-1 secretion and growth restriction caused by low-protein diets can be attributed to decreased plasma AA concentration. Evidence demonstrated that several AAs (e.g., arginine, leucine, and isoleucine) play important roles in increasing IGF-1 secretion and maintaining growth performance [14, 15]. Therefore, hepatocytes cultured with different AA concentrations are good *in vitro* model to study protein nutrition [10, 16].

Although the dietary CP or plasma AA concentration is crucial for hepatic IGF-1 expression and secretion, the intracellular mechanism underlying the role of AA in the IGF system function should be investigated. The mammalian target of rapamycin (mTOR) is one of the most important intracellular sensors of AA during cell growth and autophagy. The sensor regulates post-transcriptional protein production by activating the eukaryotic initiation factor 4E-binding protein 1 (4E-BP1) and the 70 kDa ribosomal protein S6 (p70 S6) kinase [17]. Previous studies indicated that mTOR inhibition increased casein kinase 2 activity and IGFBP-1 hyperphosphorylation in the fetal liver [18]. However, the role of the mTOR signaling pathway in AA induced IGF-1 expression and secretion remains unclear.

To describe the underlying mechanisms in dietary protein or AA induced IGF-1 secretion in liver, our study identified the signaling pathways in liver in response to protein diet by RNA-seq. Primary porcine hepatocytes and HepG2 were cells cultured in different AA concentrations and used as in vitro models to verify the role of the candidate signaling pathway in AA-induced hepatic IGF-1 secretion. Our study provided better understanding of the AA regulation of hepatic IGF-1 secretion. The findings will be valuable for pig production with the use of a low-protein dietary.

## Materials and methods

GW9662, troglitazone, ethylenediaminetetraacetic acid, dexamethasone, collagenase IV, Williams’ Medium E, porcine GH, human insulin, and 20 kinds of AAs were purchased from Sigma-Aldrich (St. Louis, MO, USA). Rapamycin was purchased from LC Laboratories (Massachusetts, USA). Penicillin–streptomycin, fetal bovine serum, high glucose Dulbecco's modified Eagle’s medium (DMEM), and hepatozyme-SFM were obtained from Life Technologies (Invitrogen, Carlsbad, CA, USA). The AA-free medium was procured from Jiang Lai Bio-Technology Co., Ltd. (Shanghai, China). Human GH was obtained from Abaier Bio-Technology Co., Ltd. (Shenzhen, China). HepG2 cells (ATCC) were purchased from Beijing zhongyuan Co., Ltd. (Beijing, China).

### Ethics statement

All animal procedures were approved by the Institutional Animal Care and Use Committee (IACUC) of the South China Agricultural University (SCAU-AEC-2010-0416).

### Animal and samples collection

A total of 12 Duroc × Landrace × Large White crossbred barrows (age, 28 d; initial weight = 9.57 ± 0.64 kg; male) were randomly assigned in two treatments and received 14% (low-protein level, LP) or 20% (control protein level, CP) crude dietary protein. The experimental diets (S1 Table) were based on corn and soybean meal. Barrows were included in the 5 days of pre-feeding before starting the experiment. The barrows had free access to water and feed. After 30 days, barrows were immediately killed after electrical stunning, and serum was collected and stored at -20°C. Liver tissue samples were collected and stored at -80°C.

### Cell culture and treatment

The 5-day-old male piglets were perfused. The experiments were approved by the IACUC of the South China Agricultural University (SCAU-AEC-2010-0416). The piglets used for hepatocyte collect were under euthanized by sodium pentobarbital injection (50 mg/kg). Hepatocytes were isolated and purified according to the two-step procedures [19–21]. The phosphate buffered saline (PBS) perfusion fluid containing 5 mM ethylenediaminetetraacetic acid was used to remove red blood cells in the liver. The perfusion medium is PBS buffered with 0.4 mg/mL collagenase IV. The liver was removed and all cells were filtered. Low-speed centrifugation at 50× g for 5 min was performed to remove the non-hepatocytes. Hepatocytes were seeded on 6-well plates at a cell density of 1×10^5^ cells/cm^2^. Cells were maintained with Williams’ Medium E, which is composed of 10% fetal bovine serum and 1% penicillin-streptomycin. After the cells reached 70%–80% confluence, the cells were incubated with the medium supplemented with physiological concentrations of 1× and 4× AA for 24 h. The physiological concentrations of AA in the AA-complete medium were: 18.775 mg/L glycine, 31.175 mg/L alanine, 21.2 mg/L serine, 23.8 mg/L threonine, 9.075 mg/L cystine, 11.175 mg/L methionine, 7.3 mg/L glutamine, 2.625 mg/L asparagine, 11.025 mg/L glutamic acid, 6.6 mg/L aspartic acid; 29.275 mg/L valine, 26.225 mg/L leucine, 19.675 mg/L isoleucine, 16.525 mg/L tyrosine, 18.2 mg/L 15.3 mg/L tryptophan, 29.225 mg/L lysine 17.4 mg/L arginine, 15.525 mg/L histidine, and 23.025 mg/L proline. The culture conditions were based on the AA concentration [22, 23], as well as those of 1 mg/L porcine GH, 100 nM human insulin, and 100 nM dexamethasone. The cells were collected and stored at -80°C.

HepG2 cell was cultured with high glucose DMEM containing 10% fetal bovine serum and 1% penicillin–streptomycin. After reaching 70%–80% confluence, the first part of HepG2 was cultured with troglitazone for 24 h. The second part of the cells was incubated with medium containing physiological concentrations of 1× and 4× AA and/or 10 μM of the PPARγ inhibitor GW9662 for 24 h and 50 nM of the mTOR inhibitor rapamycin for 48 h.

### IGF-1, albumin and urea nitrogen concentrations assays

Serum and supernatant IGF-1 concentrations were measured by RIA (NT Co., Ltd., Tianjin, China) [21]. Serum albumin and urea nitrogen concentrations were measured with commercial kits (Nanjing Jiancheng Bioengineering Institute, China).

### Total RNA isolation and reverse transcription

Standard methods and procedures of total RNA isolation and reverse transcription were used [24].

### RNA-seq

Liver samples used in RNA-seq were randomly selected from the healthy LP (*n* = 3) and CP (*n* = 3) groups. RNA-seq analysis was performed according to the manufacturer's instructions (Capital Bio Corporation, Beijing, China). The set of *Sus scrofa* transcripts was provided by ENSEMBL (ftp://ftp.ensembl.org/pub/release-73/fasta/sus_scrofa/cdna/). The sequencing reads were mapped onto the reference gene set by Bowtie [25] (Bowtie parameter:–v 3 –all–best–strata). A perl script was written to process the mapping result and generate the gene expression profile. InterPro domains [26] were interpreted by the InterProScan [27]. The livers of three pigs were selected for RNA-seq in each groups (n = 3). Transcripts per million (TPM) was used to considerate a transcript to be expressed. The general Chi^2’^ test was employed to calculate the multiple testing. Finally, transcript with a *P* value ≤ 0.01 and Fold Change ≥ 1.5 were marked to be significantly different. The Database for Annotation, Visualization, and Integrated Discovery (DAVID; http://david.abcc.ncifcrf.gov/) was applied to obtain differentially expressed genes (fold change ≥ 1.5) and to cluster genes based on their functional similarities [28].

### qPCR

A master mix contained 10 μL of SYBR Green Real-time PCR Master Mix (Toyobo Co., Ltd., Osaka, Japan), 1 μL of cDNA, 8 μL of double-distilled water, and 1 μL each of the forward and reverse primers. PCR reactions were determined with an Mx3005p instrument (Stratagene, La Jolla, CA, USA). All relative expression levels of genic mRNA were quantified by the 2^-ΔCT^ method. Porcine β-actin and human GAPDH were used as endogenous control genes.

### Western blot analysis

Standard methods for total protein extraction from cell cultures and Western blot analysis were used [29]. Blots were probed with primary antibodies, including rabbit anti-GAPDH (1:2000; Bioss), rabbit anti-IGF-1 A (1:1000; Proteintech), rabbit anti-phospho-mTOR (Ser2448) (1:1000; CST), rabbit anti-mTOR (1:2000; CST), rabbit anti-phospho-PPARγ (Ser112) (1:500; Santa Cruz), goat anti AP2 (1:1000; Santa Cruz) and rabbit anti PPARγ (1:1000; CST). Primary antibody incubation was performed at 4°C overnight, followed by incubation with the goat anti-rabbit or rabbit anti-goat antibody (1:50,000; Bioss) for 1 h at room temperature. A FluorChem M Fluorescent Imaging System (Protein Simple, Santa Clara, CA, USA) was used to measure protein expression. GAPDH was used as the endogenous control gene.

### Immunoprecipitation

A previously described immunoprecipitation (IP) procedure [30] was used with some modifications. After lysis, the cell lysate (200–500 μg total protein) was precleaned with 50 μL of Protein A+G Agarose, incubated for 10 min at 4°C, and centrifuged at 12,000×g for 15 min at 4°C. The anti-PPARγ antibody (2 μg/10 μL) was added and the mixture was incubated overnight at 4°C. Subsequently, 100 μL of Protein A+G Agarose were added and the mixture was incubated for 12 h at 4°C. The pellets were obtained by centrifugation (12,000×g for 5 min, 4°C) and thoroughly washed thrice with PBS. The pellets were dissolved in 60 μL of the electrophoresis sample buffer by Vortex suspension precipitation and subjected to Western blot assays after denaturation.

### Statistical analysis

Data are presented as means ± standard error of the means (SEM). Statistical analysis was performed with SPSS 18.0 (Chicago, IL, USA). One-way ANOVA was used for the dose effects of troglitazone. Mean differences were determined using the *t*-test followed by Fisher’s least significant difference. A confidence level of *P* < 0.05 was considered statistically significant.

## Results

### Effects of dietary protein on the serum indices and IGF expression in porcine liver

**Fig 1:**
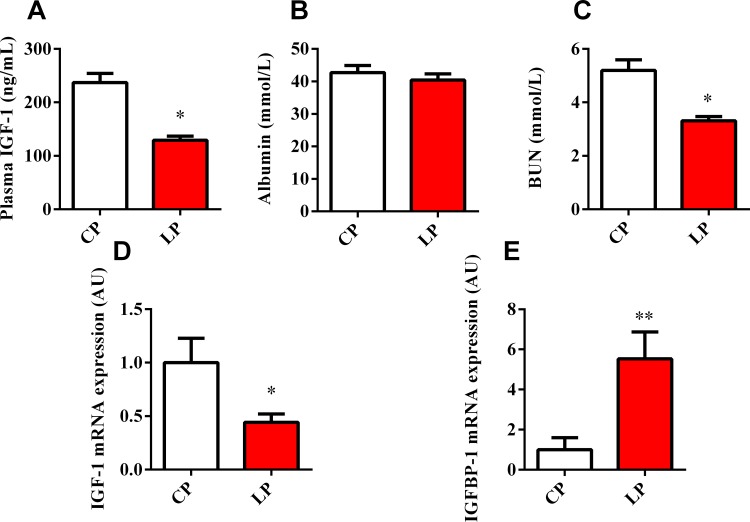
Effects of dietary protein regulated the serum index and IGF expression in porcine liver. Serum IGF-1 (A), albumin (B), and urea nitrogen (C) levels were detected in 63 day-old piglets (*n* = 6) fed with 20% crude protein diet (CP) and 14% crude protein diet (LP) using commercial kits. Total RNA was harvested and analyzed by qPCR for IGF-1 (D) and IGFBP-1 (E) mRNA expression in liver tissue (*n* = 6). Data represent the mean ± SEM. * *P* < 0.05, ** *P* < 0.01 vs. LP.

To investigate the effects of dietary protein on IGF-1 secretion, we measured the serum IGF-1 levels in pigs fed with 14% and 20% dietary protein. The results showed that the IGF-1 concentration in the low-protein (LP) group was approximately 50% that of the control protein (CP) group (*P* < 0.05; Fig 1A). Furthermore, the LP group demonstrated a lower blood urea nitrogen (BUN) concentration than the CP group (Fig 1C), whereas the serum concentration of albumin was comparable between groups (Fig 1B). Given the results with RIA, qPCR revealed the significant decrease of IGF-1 mRNA expression in the livers of the LP group compared with that of the CP group (*P* < 0.05; Fig 1D). By contrast, the mRNA expression of IGFBP-1 was significantly elevated in the LP group (*P* < 0.01; Fig 1E).

### Effects of dietary protein on transcript expression in the livers and the related differentially-expressed genes

**Fig 2:**
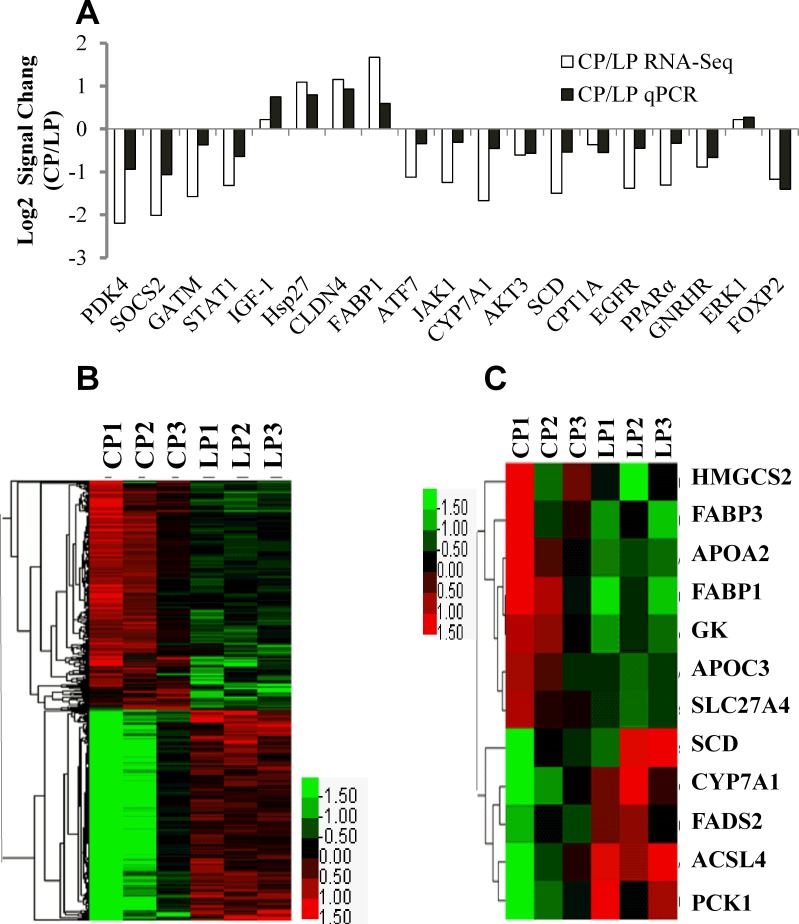
Validation of microarray results by qPCR and hierarchical cluster analysis of differentially expressed genes. (A) Comparison of expression ratios (log 2, *y*-axis; genes, *x*-axis) measured by qPCR and microarray in the 19 selected genes. Ratios by microarray and qPCR were averaged for triplicates. (B) The levels of differentially expressed genes were calculated by log2 and compared between 20% crude protein diet (CP, *n* = 3) and 14% crude protein diet (LP, *n* = 3) groups. (C) Differentially expressed genes in PPAR signaling pathway. The red color denotes high expression, whereas the green color indicates low expression.

RNA-seq technology was applied to explore the transcription factors and/or signaling pathways involved in dietary protein-induced IGF-1 secretion and IGF-1 mRNA expression. RNA-seq results revealed a total of 23,348 transcripts expressed in the livers of LP and CP (Table 1). The total number of differentially-expressed transcripts was 1319. Only 667 transcripts are upregulated and 652 expressed are downregulated based on the 1.5-fold change (*P* ≤ 0.01; ratio ≥ 1.5 or ratio ≤ 0.67). qPCR was performed on 19 randomly selected differentially-expressed genes to further validate the RNA-seq data. The analysis demonstrated that all the selected genes had a concordant direction of the fold-change between RNA-seq and qPCR (Fig 2A).

**Table 1:** Statistics of transcript expression.

Class	#	%
Total transcripts	27545	100
Expressed transcripts	23348	84.76
Expressed in CP	22374	95.83
Expressed in LP	22839	97.82
Expressed both	21865	93.65
Expressed only in CP	509	2.18
Expressed only in LP	974	4.17
Different expressed transcripts in CP/LP (p ≤ 0.01; ratio ≥ 1.5 or ratio ≤ 0.67)	Total #	1319
	Up #	667
	Down #	652

NOTE: # indicates the number of transcripts; % indicates the percentage. The livers of piglets fed with 20% crude protein diet and 14% crude protein diet are represented by CP and LP, respectively (*n* = 3).

### Identification of the signaling pathways responsive to dietary protein levels by DAVID analysis

We performed hierarchical cluster analysis to estimate the differentially-expressed transcripts in the samples (Fig 2B). Moreover, the genes of differentially-expressed transcripts were recognized by DAVID and subsequently clustered into 5 distinct functional groups (*P* < 0.05; Table 2). Five significantly-enriched pathways were associated with metabolism in KEGG-pathway, specifically the “oxidative phosphorylation”, “ribosome”, “gap junction”, “PPAR signaling pathway”, and “focal adhesion” pathways. We also used hierarchical cluster analysis to investigate the 12 differentially expressed genes in the PPAR signaling pathway (Fig 2C).

**Table 2:** Major enrichment pathways recognized by DAVID.

KEGG-pathway	Count	%	*P*-Value
Oxidative phosphorylation	28	0.244349	4.64E-07
Ribosome	17	0.148355	4.67E-04
Gap junction	15	0.130901	0.004789
PPAR signaling pathway	12	0.104721	0.010704
Focal adhesion	24	0.209442	0.020207

The differentially expressed genes were significantly enriched in different pathways according to the analysis by DAVID KEGG-pathway.

### Effects of AA on the expression of IGF-1, IGFBP-1 and PPARγ in porcine primary hepatocytes and HepG2 cells

**Fig 3:**
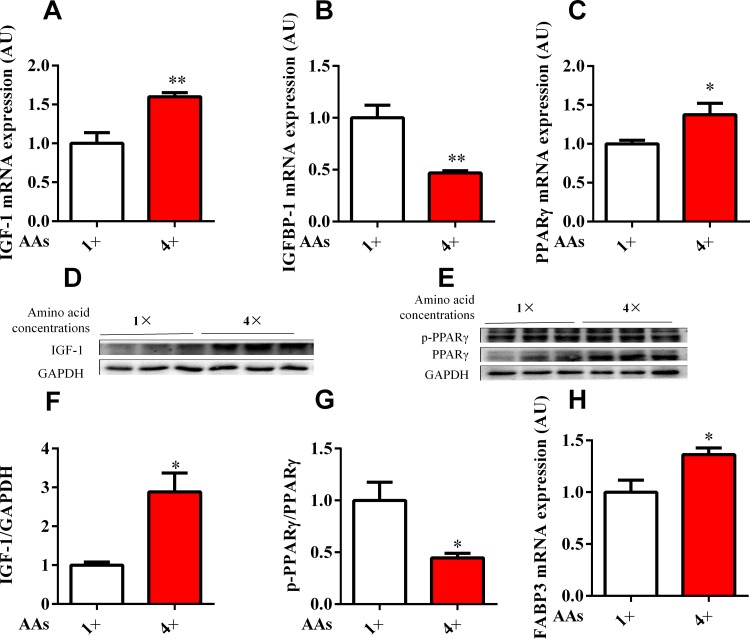
Effects of AA regulated the IGF-1, IGFBP-1, and PPARγ expression in porcine primary hepatocytes. Porcine primary hepatocytes in media with standard (1×) and four fold (4×) physiological AA concentrations were cultured for 24 h. Cellular mRNAs isolated from each treatment were subjected to qPCR analyses (*n* = 6). (A–C) IGF-1 (A), IGFBP-1 (B), PPARγ (C) and FABP3 (H) mRNA expression relative to β-actin in porcine primary hepatocytes. (D–G) The protein expression level of IGF-1 and PPARγ were assessed using Western blot. All results contain three replicates (*n* = 3). The results are expressed as mean ± SEM. * *P* < 0.05, ** *P* < 0.01 compared with cells treated with the standard (1×) group.

**Fig 4:**
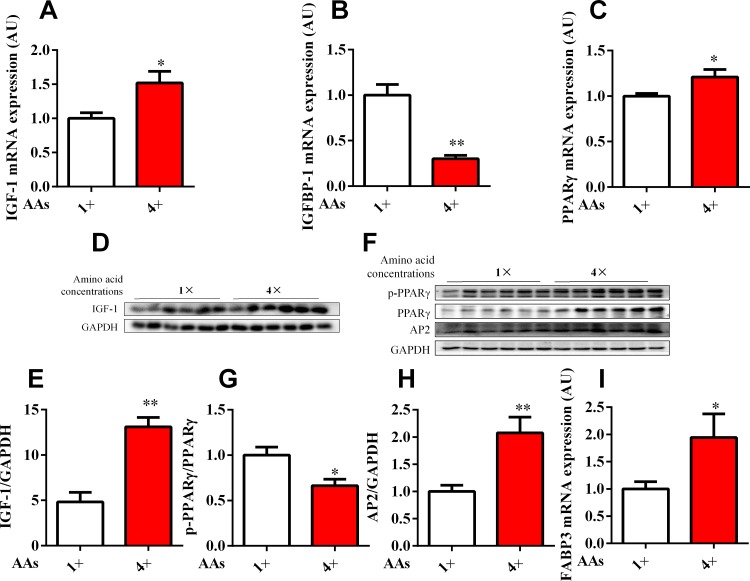
Effects of AA regulated the expression of IGF-1, IGFBP-1 and PPARγ in HepG2 cells. HepG2 cells were culture in media with the standard (1×) and 4 times (4×) physiological AA concentrations for 24 h. IGF-1 (A), IGFBP-1 (B), PPARγ (C) and FABP3 (I) mRNA expression was assessed by qPCR. (D–H) Protein expression levels of IGF-1, GAPDH, PPARγ, p-PPARγ and AP2 were assessed by Western blot analysis. All results were obtained from three replicates (*n* = 6). Results were expressed as mean ± SEM. * *P* < 0.05, ** *P* < 0.01 vs. cells treated with the standard group.

The porcine primary hepatocytes and HepG2 cell line were exposed to physiological concentrations of 1× and 4× AA to mimic the in vitro model for high protein. The results showed that the mRNA expression of IGF-1 (Figs 3A and 4A) and PPARγ (Figs 3C and 4C) and its target gene, FABP3 (Figs 3H and 4I) in hepatocytes were significantly (*P* < 0.05) upregulated in response to higher AA levels, whereas IGFBP-1 was inhibited by physiological concentrations of 4× AA (Figs 3B and 4B). The Western blot data further confirmed that IGF-1 (Figs 3F and 4E) and PPARγ (Figs 3E and 4F) protein expression were consistent with the mRNA levels. The p-PPARγ level was dramatically reduced by physiological concentrations of 4× AA in porcine primary hepatocytes and HepG2 cells (Figs 3G and 4G). In addition, AP2 protein, another PPARγ target genes was also significant enhanced in 4× physiological concentrations of AA in HepG2 cells (Fig 4H). Therefore, PPARγ activity was enhanced with increasing AA concentration.

### Effects of AA on IGF-1 and IGFBP-1 mRNA expression mediated by PPARγ

**Fig 5:**
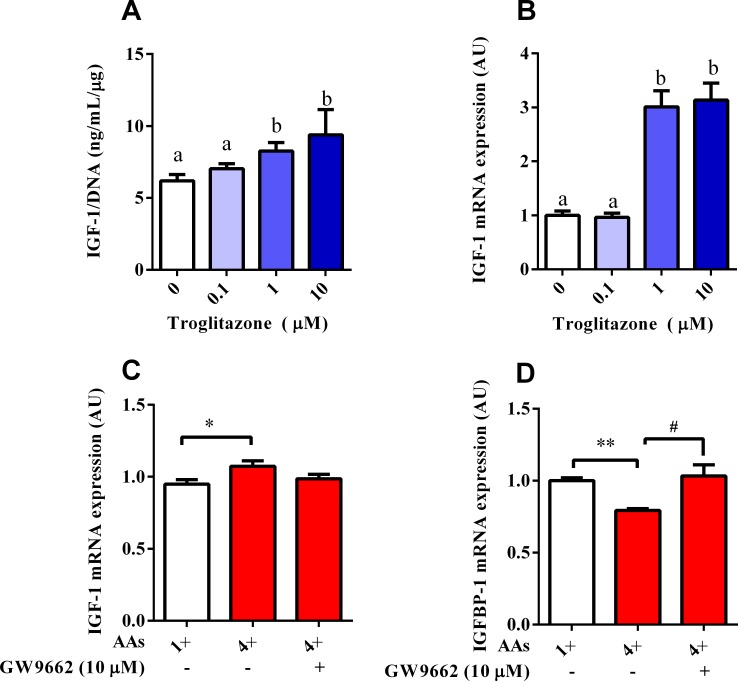
Effects of AA on IGF-1 and IGFBP-1 mRNA expression were mediated by PPARγ. IGF-1 secretion (A) and IGF-1 mRNA expression (B) were measured after treatment with the PPARγ agonist troglitazone. Values with different letters were significantly different (*P* < 0.05, *n* = 6). (C and D) Cells treated with HepG2 in media with 1× or 4× physiological AA concentrations, which contained 10 μM of the PPARγ inhibitor GW9662. IGF-1 and IGFBP-1 mRNA expression were analyzed by qPCR. *P < 0.05 (*n* = 6). Results were expressed as mean ± SEM.

Co-treatment with the PPARγ agonist and antagonist was used to determine the involvement of PPARγ in AA-induced IGF-1 expression. The IGF-1 concentrations were determined by RIA with an intraassay CV of less than 10% and an interassay CV of less than 8%. The results demonstrated that the PPARγ agonist (troglitazone) increased the IGF-1 content of the culture medium in a dose-dependent manner (Fig 5A). The mRNA level of IGF-1 was also remarkably (*P* < 0.05) elevated by troglitazone (1 and 10 μM; Fig 5B). The PPARγ antagonist (GW9662) eliminated the effects of 4× AA on the mRNA expression of IGF-1 (Fig 5C) and IGFBP-1 (Fig 5D), respectively. These results indicated that PPARγ was involved in the regulation of IGF-1 in response to different AA concentrations.

### mTOR involvement in the AA-induced activation of PPARγ

**Fig 6:**
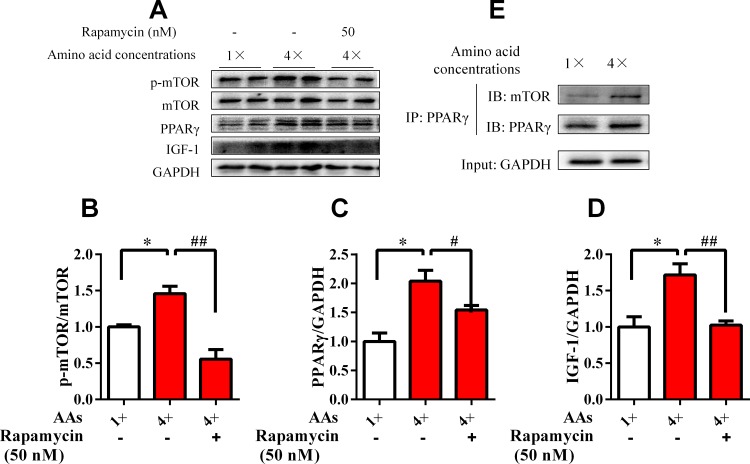
mTOR was involved in the AA-induced activation of PPARγ. HepG2 cells were cultured in media with 1× and 4× physiological AA concentrations for 48 h. One fraction of the total protein was used to determine the total and phosphorylated levels of the mTOR (A and B), GAPDH, PPARγ (C), and IGF-1 (D) proteins by Western blot analysis. All results contained three replicates (*n* = 3). The other total protein extracts were analyzed by immunoprecipitation (IP) with anti-PPARγ (E) capture antibodies. Data were expressed as the mean ± SEM. Values with different letters were significantly different (*P* < 0.05).

mTOR is an endogenous sensor for AAs. To delineate the role of mTOR in AA-induced PPARγ activation, HepG2 cells cultured with 4× AA were co-treated with mTOR inhibitor rapamycin (50 nM). Results showed that rapamycin completely blocked the mTOR activity (p-mTOR/mTOR; Fig 6B). Therefore, the AA-induced PPARγ and IGF-1 expression were effectively reversed by rapamycin (Fig 6C and 6D). We further analyzed the protein–protein interaction between mTOR and PPARγ by Co-immunoprecipitation. Physiological concentrations of 4× AA promoted the interaction between mTOR and PPARγ protein (Fig 6E). In summary, these findings indicated that the interaction of mTOR and PPARγ is involved in AA-induced activation of PPARγ and IGF-1 expression.

## Discussion

Low dietary CP supplemented with essential AAs (EAA) is an effective strategy to reduce nitrogen excretion and increase the dietary protein efficiency in pig production [31]. However, CP reduction greater than 4% often restricts growth as the serum IGF-1 concentration decreases [11, 32]. Our results revealed that serum IGF-1 concentrations and liver IGF-1 gene expression in the low-protein group were significantly reduced compared with the control protein group, which is consistent with previous studies [11, 33, 34]. The low-protein diet also significantly reduced the growth performance index of piglets. The growth performance and serum IGF-1 level were difficult to recover by supplementing with 4 EAAs when dietary CP was reduced by 6%. Therefore, we studied the transcriptome differences by RNA-seq to reveal the underlying mechanism of protein- or AA-induced hepatic IGF-1 expression and secretion.

The general amino acid control non-derepressible 2 (GCN2) has been widely cited as a specific sensor for deprivation of amino acids [35]. It has been revealed that amino acid starvation increase the phosphorylation of GCN2 and then activate the downstream molecular, including eIF2a and ATF4 [36, 37]. However, neither GCN2, nor eIF2a and ATF4 were found differentially expressed based on our RNA-seq analysis data. It seems that experimental models may interpret this unexpected phenomenon. Most of previous publications identified the role of GCN2 kinase in sensing amino acids limitation base on specific amino acids deficient model *in vitro* [37–40] and *in vivo* [41–43]. In contrast, our *in vitro* study only changed the total content of AA in normal physiological limits without any AA deprivation. In addition, several crucial AA, such as lysine, threonine, tryptophan and methionine were also balanced between 20% and 14% dietary protein feed in our in vivo study. Therefore, those present evidences supported that GCN2 signaling pathway may not the key sensor for AA change within physiological range.

Our data also demonstrated that PPAR is a prospective signaling pathway in response to lower levels of dietary protein. PPARs are a group of nuclear receptor proteins that function as transcription factors that regulate gene expression, with essential roles in the regulation of cellular differentiation [44], development [45], and metabolism [46]. Our in vitro studies revealed that 4× AA significantly enhanced the gene expression of PPARγ and IGF-1 compared with 1× AA in porcine primary hepatocytes and human HepG2 cells. In addition, PPARγ inhibition eliminated the effects of 4× AA on the mRNA expression of IGF-1 and IGFBP-1, respectively. These evidence suggested that PPARγ is crucial for AA-induced hepatic IGF-1 expression and secretion. However, a previous study showed that the PPARγ agonist (rosiglitazone) significantly reduced serum IGF-1 conentrations accompanied by markedly suppressed IGF-1 transcription in the liver [47]. The discrepancy for IGF-I gene expression in response to the activation of PPARγ may attribute to the different sequence of IGF-I promoter region between human and murine species. Similarly, some other PPARs target gene, such as apolipoprotein A-I and glyoxylate reductase/hydroxypyruvate reductase, are also differentially regulated by PPARs between rat and human [48–50]. Therefore, our study demonstrated a novel role of PPARγ for IGF-1 regulation in porcine and human systems as opposed to murine species.

The endogenous ligands of PPARs are mainly long-chain fatty acids, such as docosahexaenoic acid, eicosapentaenoic acid, and eicosanoids (leukotriene B4 and prostaglandin PGJ2) [51]. Natural and synthetic ligands, such as thiazolidinediones, are widely used [44]. mTOR is an important regulator that combines AA availability with cell growth and autophagy [17]. This study showed that high concentrations of AAs increased the protein levels of p-mTOR, PPARγ, and IGF-1, as well as induced protein–protein interaction between mTOR and PPARγ in HepG2 cells. Furthermore, the PPARγ levels enhanced by 4× AA could be decreased by rapamycin (the mTOR inhibitor) in HepG2 cells. This data is consistent with the previous finding that ghrelin stimulated hepatic lipogenesis was also mediated by activating the mTOR-PPARγ signaling pathway in hepatocytes [52]. Since raptor is a critical component of TORC1, the strength of the association between mTOR and raptor is regulated by nutrients that regulate the mTORC1 pathway [53]. Therefore, the complex of mTOR and raptor may necessary to bind and active PPARγ. Together, these evidences strongly supported that AA-induced hepatic IGF-1 expression and secretion are mediated by mTOR/PPARγ pathway.

## Conclusion

We provided evidence that PPARγ is involved in the regulation of hepatic IGF-1 secretion and gene expression in response to the protein diet. PPARγ plays an important role in the AA regulation of IGF-1 expression in hepatocytes. Therefore, the current understanding of the molecular mechanisms of liver IGF-1 secretion in response to dietary protein or AAs is expanded.

## Supporting information

S1 Fig Western Blots. (PDF)

S1 Table Composition of experimental diets for barrows. (PDF)

S2 Table Up-regulated and down-regulated transcripts in liver. (DOCX)
