# Supplementary figures and images for: Dietary protein-induced hepatic IGF-1 secretion mediated by PPARγ activation
Source: PLoS One. 2017 Mar 3;12(3):e0173174. doi: 10.1371/journal.pone.0173174 (PMC5336265; doi:10.1371/journal.pone.0173174)

S1 Fig. Western Blots

Fig 4

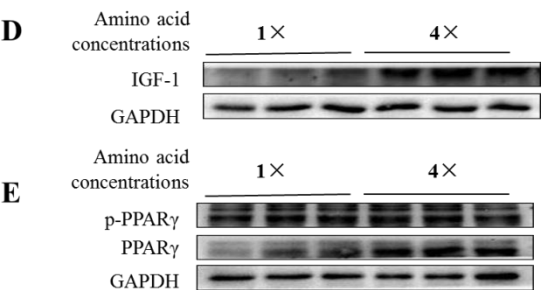

Fig 5

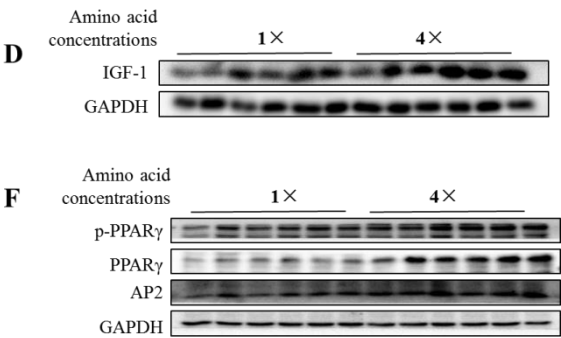

Fig 7

**A**

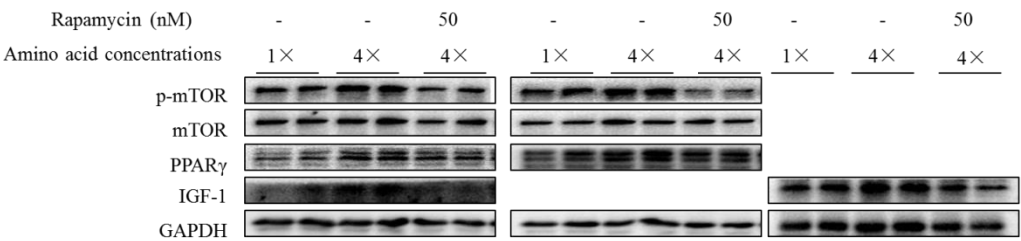

**E**

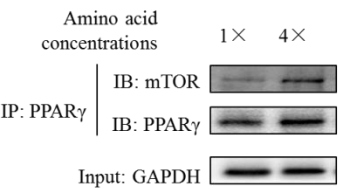

Supplement: S1 Fig — (PDF) [file pone.0173174.s001.pdf]
