# Supplementary material for: Dietary protein-induced hepatic IGF-1 secretion mediated by PPARγ activation
Source: PLoS One. 2017 Mar 3;12(3):e0173174. doi: 10.1371/journal.pone.0173174 (PMC5336265; doi:10.1371/journal.pone.0173174)
Supplement: S1 Table — (PDF) [file pone.0173174.s002.pdf]

S1 Table. Composition of experimental diets for barrows

Item	Dietary crude protein concentration, %	
	14	20
Ingredients, %		
*Digestible energy（MJ/kg）	14.60	14.60
Crude protein	14.14	20.27
Lysine	1.26	1.26
Methionine and cysteine	0.63	0.62
Threonine	0.76	0.76
Tryptophan	0.20	0.20
Arginine	0.71	1.09
Histidine	0.30	0.44
Isoleucine	0.46	0.71
Leucine	1.11	1.52
Phenylalanine	0.56	0.81
Valine	0.54	0.72
Calcium	0.70	0.69
Phosphorus	0.53	0.57
Essential amino acids	6.29	7.91
Non-essential amino acids	6.84	9.74
Essential amino acids /non-essential amino acids	0.90	0.80
