# Supplementary material for: Dietary protein-induced hepatic IGF-1 secretion mediated by PPARγ activation
Source: PLoS One. 2017 Mar 3;12(3):e0173174. doi: 10.1371/journal.pone.0173174 (PMC5336265; doi:10.1371/journal.pone.0173174)
Supplement: S2 Table — (DOCX) [file pone.0173174.s003.docx]

**S2 Table. Up-regulated and down-regulated transcripts in liver**

**Up-regulated transcripts in liver**

| **Transcripts ID** | **Ratio** | **P Value** | **Transcripts** |
| --- | --- | --- | --- |
| ENSSSCT00000024670 | 1.578 | 0.003598205 | CMBL |
| ENSSSCT00000023046 | 1.88 | 0.000140472 | PPIF |
| ENSSSCT00000024184 | 1.755 | 0.000620465 | NDUFA11 |
| ENSSSCT00000027628 | 1.535 | 0.003336098 | MMACHC |
| ENSSSCT00000029635 | 1.716 | 0.000709671 | AP2S1 |
| ENSSSCT00000000025 | 1.505 | 0.008638601 | PARVB |
| ENSSSCT00000008867 | 1.791 | 0.000801365 | CHCHD5 |
| ENSSSCT00000025008 | 1.74 | 0.002054238 | CHCHD5 |
| ENSSSCT00000025595 | 2.024 | 0.000234359 | ATP5J2 |
| ENSSSCT00000036614 | 1.558 | 0.008254607 | CD63 |
| ENSSSCT00000029362 | 1.647 | 0.001551878 | ATPAF2 |
| ENSSSCT00000000319 | 1.576 | 0.00700756 | PPP1R1A |
| ENSSSCT00000005854 | 2.05 | 0.000251682 | TPM1 |
| ENSSSCT00000012912 | 1.701 | 0.002535711 | FETUB |
| ENSSSCT00000019103 | 1.697 | 0.000830656 | PHB |
| ENSSSCT00000001531 | 1.571 | 0.004101103 | NFKB1 |
| ENSSSCT00000003448 | 1.831 | 0.000948141 | TMEM160 |
| ENSSSCT00000008896 | 1.841 | 0.000209011 | FAHD2A |
| ENSSSCT00000017099 | 2.346 | 5.75E-06 | ACMSD |
| ENSSSCT00000000756 | 1.959 | 0.000149899 | GAPDH |
| ENSSSCT00000005263 | 1.84 | 0.000511873 | GCHFR |
| ENSSSCT00000017530 | 1.628 | 0.001475765 | NDUFB3 |
| ENSSSCT00000003359 | 1.562 | 0.00317708 | PAFAH1B3 |
| ENSSSCT00000003367 | 1.827 | 0.000755723 | PAFAH1B3 |
| ENSSSCT00000004329 | 1.568 | 0.003856374 | PRDX1 |
| ENSSSCT00000002815 | 1.695 | 0.003436684 | HSP90AA1 |
| ENSSSCT00000014708 | 1.591 | 0.002127762 | LSM7 |
| ENSSSCT00000018607 | 2.133 | 8.31E-05 | ANXA6 |
| ENSSSCT00000032692 | 1.724 | 0.001188941 | LOC465727 |
| ENSSSCT00000033591 | 1.554 | 0.003225079 | LOC465727 |
| ENSSSCT00000008782 | 1.628 | 0.002145136 | NUBP2 |
| ENSSSCT00000012470 | 1.725 | 0.001955418 | C3orf54 |
| ENSSSCT00000001079 | 2.213 | 0.00039381 | C6orf129 |
| ENSSSCT00000002936 | 1.573 | 0.003122731 | TRAPPC2L |
| ENSSSCT00000000979 | 1.576 | 0.002117181 | SMF |
| ENSSSCT00000028155 | 1.612 | 0.002311001 | EEF1B |
| ENSSSCT00000007619 | 1.749 | 0.000834016 | EEF1B |
| ENSSSCT00000001854 | 1.86 | 0.008985427 | DNPH1 |
| ENSSSCT00000001861 | 2.097 | 0.001448126 | DNPH1 |
| ENSSSCT00000011034 | 2.189 | 2.33E-05 | CHCHD10 |
| ENSSSCT00000028123 | 1.533 | 0.005259851 | RPS15 |
| ENSSSCT00000019497 | 1.67 | 0.005821285 | VMO1 |
| ENSSSCT00000028028 | 1.616 | 0.001522263 | TIM50 |
| ENSSSCT00000013071 | 2.193 | 0.000118285 | ATP5G3 |
| ENSSSCT00000023954 | 1.615 | 0.001731445 | MRPL45 |
| ENSSSCT00000029868 | 2.064 | 0.000253062 | MRPL55 |
| ENSSSCT00000018385 | 1.671 | 0.00081906 | ATP5H |
| ENSSSCT00000024892 | 1.518 | 0.007003826 | RPS2 |
| ENSSSCT00000026560 | 1.5 | 0.007739379 | NUDT5 |
| ENSSSCT00000011065 | 1.76 | 0.000731411 | FAM128B |
| ENSSSCT00000002914 | 1.61 | 0.002077946 | GSTA1 |
| ENSSSCT00000023414 | 1.992 | 0.000163573 | TRA16 |
| ENSSSCT00000002915 | 1.772 | 0.000268144 | GSTA4 |
| ENSSSCT00000006287 | 1.527 | 0.002504576 | UCKL1 |
| ENSSSCT00000028130 | 1.578 | 0.003318115 | RpS17 |
| ENSSSCT00000018659 | 1.723 | 0.000809658 | Chmp6 |
| ENSSSCT00000009521 | 1.833 | 0.002378721 | DOK7 |
| ENSSSCT00000023513 | 1.546 | 0.003013192 | ITGB4BP |
| ENSSSCT00000032340 | 1.7 | 0.001321682 | TMEM258 |
| ENSSSCT00000008740 | 1.921 | 0.002412737 | HBM |
| ENSSSCT00000025201 | 1.586 | 0.00638264 | HRAS1 |
| ENSSSCT00000005034 | 1.855 | 0.000712261 | PPIB |
| ENSSSCT00000034348 | 1.841 | 0.007224202 | HBA1 |
| ENSSSCT00000027777 | 1.577 | 0.002371456 | GRPEL1 |
| ENSSSCT00000000053 | 1.623 | 0.001519024 | SMDT1 |
| ENSSSCT00000000296 | 1.58 | 0.001892603 | TARBP2 |
| ENSSSCT00000005187 | 1.938 | 0.000250216 | HYPK |
| ENSSSCT00000028385 | 1.534 | 0.003582029 | ZC3HC1 |
| ENSSSCT00000017033 | 2.081 | 0.000213091 | SEC61G |
| ENSSSCT00000015634 | 1.734 | 0.002226737 | CXCL14 |
| ENSSSCT00000007907 | 1.602 | 0.002660398 | ID1 |
| ENSSSCT00000024149 | 1.676 | 0.000932452 | MPDU1 |
| ENSSSCT00000000089 | 1.946 | 0.000256113 | RPS19BP1 |
| ENSSSCT00000015639 | 1.678 | 0.001230065 | LECT2 |
| ENSSSCT00000000746 | 2.041 | 4.60E-05 | TPI1 |
| ENSSSCT00000032650 | 1.707 | 0.001384956 | IL17RA |
| ENSSSCT00000006406 | 2.499 | 8.35E-06 | EGFL7 |
| ENSSSCT00000013078 | 1.506 | 0.00539872 | RPL24 |
| ENSSSCT00000001759 | 2.547 | 8.33E-05 | CCDC167 |
| ENSSSCT00000025194 | 1.731 | 0.001150921 | taf10 |
| ENSSSCT00000000122 | 1.506 | 0.003636539 | Polr2e |
| ENSSSCT00000028233 | 1.586 | 0.007649777 | PPP1R1A |
| ENSSSCT00000006403 | 1.568 | 0.004718877 | PHPT1 |
| ENSSSCT00000018580 | 1.577 | 0.007563774 | Mrpl22 |
| ENSSSCT00000008772 | 1.754 | 0.00148172 | FAM173A |
| ENSSSCT00000006421 | 1.565 | 0.002789214 | Tmem141 |
| ENSSSCT00000027931 | 1.565 | 0.002640671 | COX5A |
| ENSSSCT00000005075 | 1.588 | 0.004225107 | RPS15A |
| ENSSSCT00000010964 | 1.632 | 0.005381379 | MTP18 |
| ENSSSCT00000026822 | 1.766 | 0.001228306 | RUVBL1 |
| ENSSSCT00000033244 | 1.503 | 0.000539024 | HAGHL |
| ENSSSCT00000015701 | 1.559 | 0.002885802 | NDUFA2 |
| ENSSSCT00000011024 | 2.032 | 0.000135212 | DDT |
| ENSSSCT00000012218 | 1.765 | 0.000959066 | MCTS1 |
| ENSSSCT00000010014 | 1.638 | 0.0020809 | DC2 |
| ENSSSCT00000003218 | 1.606 | 0.001740626 | PRODH2 |
| ENSSSCT00000033011 | 1.789 | 0.001308969 | FDX1L |
| ENSSSCT00000018719 | 1.661 | 0.003938236 | TEN1 |
| ENSSSCT00000032731 | 1.531 | 0.007761993 | MAP2K2 |
| ENSSSCT00000014855 | 1.65 | 0.001008666 | MARCH2 |
| ENSSSCT00000025493 | 1.53 | 0.005334524 | MID1IP1 |
| ENSSSCT00000033394 | 1.614 | 0.003199588 | TRAPPC9 |
| ENSSSCT00000018238 | 1.509 | 0.003946017 | BLVRA |
| ENSSSCT00000029962 | 1.992 | 0.000204104 | OPLAH |
| ENSSSCT00000015054 | 1.897 | 0.000290324 | Ndufb7 |
| ENSSSCT00000010473 | 1.713 | 0.000756993 | GRTP1 |
| ENSSSCT00000004917 | 2.962 | 1.94E-05 | PRSS35 |
| ENSSSCT00000014119 | 1.762 | 0.000542054 | POLD4 |
| ENSSSCT00000011581 | 1.978 | 0.000162638 | ARL3 |
| ENSSSCT00000001070 | 1.524 | 0.006199419 | ALG12 |
| ENSSSCT00000030615 | 1.55 | 0.004467702 | ENY2 |
| ENSSSCT00000018239 | 1.579 | 0.005960183 | CG044 |
| ENSSSCT00000000128 | 2.079 | 4.84E-05 | GCAT |
| ENSSSCT00000004396 | 1.586 | 0.00665736 | CITED4 |
| ENSSSCT00000013753 | 1.779 | 0.000824601 | COPE |
| ENSSSCT00000006479 | 1.848 | 0.000941795 | GPT |
| ENSSSCT00000030558 | 1.611 | 0.001943429 | UBL5 |
| ENSSSCT00000008386 | 1.542 | 0.003887564 | C7orf59 |
| ENSSSCT00000003959 | 2.228 | 6.99E-06 | NR0B2 |
| ENSSSCT00000028246 | 1.639 | 0.001986155 | KHK |
| ENSSSCT00000016169 | 1.714 | 0.000843471 | STARD10 |
| ENSSSCT00000015022 | 1.696 | 0.0015142 | GADD45GIP1 |
| ENSSSCT00000022560 | 1.563 | 0.003910247 | hsRPB7 |
| ENSSSCT00000008748 | 1.751 | 0.001036146 | MRPL28 |
| ENSSSCT00000008551 | 1.52 | 0.003450254 | EFTu |
| ENSSSCT00000014158 | 1.647 | 0.001239642 | YIF1A |
| ENSSSCT00000011038 | 2.395 | 0.000141678 | IGLL1 |
| ENSSSCT00000011026 | 2.801 | 4.20E-05 | Gstt4 |
| ENSSSCT00000012835 | 1.514 | 0.005320082 | GMPS |
| ENSSSCT00000018843 | 1.55 | 0.005073436 | MYL4 |
| ENSSSCT00000000006 | 1.732 | 0.001190197 | C22orf40 |
| ENSSSCT00000019457 | 1.636 | 0.002303143 | SPNS3 |
| ENSSSCT00000003431 | 2.044 | 2.16E-05 | FOXA3 |
| ENSSSCT00000003961 | 1.683 | 0.003416586 | C1orf172 |
| ENSSSCT00000027805 | 1.5 | 0.004111101 | SND1 |
| ENSSSCT00000035419 | 1.599 | 0.001640921 | Xbp1 |
| ENSSSCT00000030292 | 1.615 | 0.006954991 | CCP1-4 |
| ENSSSCT00000036318 | 1.534 | 0.006152172 | PIK3R1 |
| ENSSSCT00000034322 | 1.634 | 0.002286004 | ILF2 |
| ENSSSCT00000032984 | 1.588 | 0.004032354 | IL11RA |
| ENSSSCT00000015041 | 1.531 | 0.004652323 | PALM3 |
| ENSSSCT00000011560 | 1.589 | 0.007732774 | NPM3 |
| ENSSSCT00000024428 | 1.866 | 0.000337931 | LTC4S |
| ENSSSCT00000000133 | 1.723 | 0.000632259 | kbl |
| ENSSSCT00000014449 | 1.688 | 0.00101156 | Ndufs3 |
| ENSSSCT00000033111 | 1.703 | 0.001528416 | FAM58A |
| ENSSSCT00000001097 | 1.688 | 0.001305716 | PSMG4 |
| ENSSSCT00000014251 | 1.513 | 0.004912738 | DNAJC4 |
| ENSSSCT00000011612 | 1.606 | 0.003055979 | GSTO1 |
| ENSSSCT00000018332 | 1.557 | 0.009811805 | RPS24 |
| ENSSSCT00000002806 | 1.962 | 0.000250681 | ANKRD9 |
| ENSSSCT00000003853 | 1.532 | 0.007190816 | SLC25A34 |
| ENSSSCT00000014252 | 2.21 | 9.55E-05 | NUDT22 |
| ENSSSCT00000003990 | 1.825 | 0.000535204 | SERINC2 |
| ENSSSCT00000027814 | 1.683 | 0.000905619 | ZMYM3 |
| ENSSSCT00000018728 | 1.873 | 0.000360023 | galK |
| ENSSSCT00000008523 | 1.618 | 0.002802901 | BCL7C |
| ENSSSCT00000000078 | 1.517 | 0.002802863 | SLC25A17 |
| ENSSSCT00000003279 | 1.559 | 0.002293842 | PSMD8 |
| ENSSSCT00000012722 | 1.506 | 0.006521355 | CHST13 |
| ENSSSCT00000026102 | 1.525 | 0.006092443 | RCN3 |
| ENSSSCT00000006494 | 1.578 | 0.004762177 | SHARPIN |
| ENSSSCT00000035128 | 1.545 | 0.003165652 | AIFM1 |
| ENSSSCT00000011685 | 1.553 | 0.007040326 | RGS10 |
| ENSSSCT00000004710 | 1.937 | 0.000592958 | RPLP1 |
| ENSSSCT00000014045 | 1.514 | 0.00219456 | DEAF1 |
| ENSSSCT00000029183 | 1.675 | 0.000921781 | MRPL40 |
| ENSSSCT00000008427 | 1.525 | 0.008064899 | ALKBH4 |
| ENSSSCT00000033187 | 1.623 | 0.001640085 | BCAP31 |
| ENSSSCT00000033957 | 1.696 | 0.000914052 | BCAP31 |
| ENSSSCT00000034466 | 1.576 | 0.002790823 | BCAP31 |
| ENSSSCT00000033724 | 1.911 | 0.000243287 | GK |
| ENSSSCT00000036515 | 1.876 | 0.00024216 | GK |
| ENSSSCT00000018735 | 1.509 | 0.004139428 | MRPS7 |
| ENSSSCT00000030896 | 1.539 | 0.002439327 | SLC25A39 |
| ENSSSCT00000008780 | 1.922 | 9.60E-05 | HAGH |
| ENSSSCT00000018205 | 1.582 | 0.00206218 | Mrps24 |
| ENSSSCT00000008784 | 1.593 | 0.002637885 | Mrps34 |
| ENSSSCT00000005627 | 1.53 | 0.003665046 | MRPL46 |
| ENSSSCT00000035384 | 1.695 | 0.001212174 | MAGED1 |
| ENSSSCT00000014675 | 1.701 | 0.000874011 | ATP5D |
| ENSSSCT00000028192 | 1.705 | 0.002339875 | AKNA |
| ENSSSCT00000000079 | 1.836 | 0.00050778 | Rpl31 |
| ENSSSCT00000019468 | 1.55 | 0.004092654 | TXNDC17 |
| ENSSSCT00000006458 | 1.624 | 0.002915583 | COMMD5 |
| ENSSSCT00000003877 | 1.525 | 0.004804081 | MRTO4 |
| ENSSSCT00000008785 | 1.605 | 0.002105771 | NDPK3 |
| ENSSSCT00000030646 | 1.683 | 0.001831538 | RPS29 |
| ENSSSCT00000025089 | 1.724 | 0.001146024 | RPS29 |
| ENSSSCT00000003666 | 2.182 | 2.35E-05 | TMEM86B |
| ENSSSCT00000006468 | 1.732 | 0.001477829 | COMMD5 |
| ENSSSCT00000003577 | 2.045 | 0.000723779 | FAM71E1 |
| ENSSSCT00000034343 | 1.586 | 0.003493514 | RPL8 |
| ENSSSCT00000033716 | 1.597 | 0.002975738 | RPL8 |
| ENSSSCT00000034388 | 1.709 | 0.002009838 | RPL8 |
| ENSSSCT00000033850 | 1.547 | 0.004839386 | RPL8 |
| ENSSSCT00000035903 | 1.52 | 0.00635199 | RPL8 |
| ENSSSCT00000017034 | 1.593 | 0.002622616 | C1orf186 |
| ENSSSCT00000008762 | 2.279 | 1.83E-05 | FAM195A |
| ENSSSCT00000000477 | 1.809 | 0.000985377 | INHBE |
| ENSSSCT00000014050 | 1.518 | 0.003927717 | RASSF7 |
| ENSSSCT00000013429 | 1.752 | 0.00274097 | UXT |
| ENSSSCT00000008352 | 1.807 | 0.000335245 | BUD31 |
| ENSSSCT00000007472 | 1.968 | 0.007588873 | GSTM5 |
| ENSSSCT00000012006 | 1.913 | 0.000170743 | IL11RA |
| ENSSSCT00000023267 | 2.182 | 0.000121831 | PRG3 |
| ENSSSCT00000008483 | 1.781 | 0.000335077 | ASL |
| ENSSSCT00000031817 | 1.728 | 0.001526848 | MRPL27 |
| ENSSSCT00000031420 | 1.514 | 0.005789179 | ZNF428 |
| ENSSSCT00000022447 | 2.445 | 1.31E-05 | APRT |
| ENSSSCT00000002941 | 2.58 | 6.13E-06 | APRT |
| ENSSSCT00000003401 | 1.711 | 0.000459642 | ETHE1 |
| ENSSSCT00000024316 | 1.643 | 0.003640777 | MRPL27 |
| ENSSSCT00000006980 | 1.592 | 0.004083078 | TSTD1 |
| ENSSSCT00000023357 | 1.727 | 0.000795585 | TIMM8B |
| ENSSSCT00000036026 | 1.574 | 0.007180825 | SIGLEC5 |
| ENSSSCT00000025869 | 1.664 | 0.001421225 | CARS |
| ENSSSCT00000001895 | 1.88 | 0.000438303 | MRPL14 |
| ENSSSCT00000018753 | 1.83 | 0.003978418 | FADS6 |
| ENSSSCT00000011308 | 1.633 | 0.001285162 | cyclophilin D |
| ENSSSCT00000015027 | 1.778 | 0.00141049 | IER2 |
| ENSSSCT00000006505 | 1.724 | 0.001355692 | RRP41 |
| ENSSSCT00000008489 | 1.564 | 0.002380105 | CHCHD2 |
| ENSSSCT00000030529 | 1.666 | 0.001018403 | C6orf125 |
| ENSSSCT00000015177 | 1.544 | 0.002753661 | CCDC124 |
| ENSSSCT00000024289 | 3.268 | 3.44E-08 | FGF23 |
| ENSSSCT00000007423 | 1.688 | 0.001003328 | RHOC |
| ENSSSCT00000002833 | 2.039 | 0.000109973 | MP68 |
| ENSSSCT00000018910 | 1.53 | 0.005208571 | TMEM101 |
| ENSSSCT00000000189 | 1.612 | 0.003359014 | FKBP11 |
| ENSSSCT00000014174 | 1.593 | 0.007572657 | FOSL1 |
| ENSSSCT00000008382 | 1.782 | 0.000187458 | AZGP1 |
| ENSSSCT00000030078 | 2.652 | 0.000238102 | PYY |
| ENSSSCT00000006507 | 1.506 | 0.004436521 | GPAA1 |
| ENSSSCT00000015158 | 1.515 | 0.005346945 | USE1 |
| ENSSSCT00000014032 | 1.525 | 0.003402913 | CHID1 |
| ENSSSCT00000002239 | 1.526 | 0.005908219 | REC8 |
| ENSSSCT00000029526 | 1.752 | 0.001589322 | GSDMB |
| ENSSSCT00000007149 | 1.567 | 0.002240489 | KRTCAP2 |
| ENSSSCT00000007150 | 1.5 | 0.008789059 | DPM3 |
| ENSSSCT00000033922 | 1.913 | 0.000110146 | NOV |
| ENSSSCT00000026713 | 1.615 | 0.001505623 | ECI1 |
| ENSSSCT00000034958 | 1.51 | 0.009866714 | MED29 |
| ENSSSCT00000015156 | 1.568 | 0.001986708 | Nr2f6 |
| ENSSSCT00000026222 | 1.968 | 0.000499748 | MRPL55 |
| ENSSSCT00000028688 | 1.853 | 0.000264092 | SERTAD1 |
| ENSSSCT00000002214 | 1.781 | 0.000980109 | Mcpt3 |
| ENSSSCT00000014037 | 1.563 | 0.004476901 | RPLP2 |
| ENSSSCT00000019496 | 1.744 | 0.001744883 | Gltpd2 |
| ENSSSCT00000026577 | 1.618 | 0.004168384 | PPCDC |
| ENSSSCT00000008251 | 1.61 | 0.002309439 | ATP5E |
| ENSSSCT00000033972 | 1.57 | 0.002951279 | ATP5E |
| ENSSSCT00000008496 | 1.869 | 0.000475884 | MRPS17 |
| ENSSSCT00000019360 | 1.716 | 0.000735272 | Tp53i13 |
| ENSSSCT00000010611 | 1.934 | 0.000941827 | RPLP1 |
| ENSSSCT00000012208 | 1.633 | 0.002121767 | MCTS1 |
| ENSSSCT00000033875 | 1.567 | 0.003202238 | SSR4 |
| ENSSSCT00000034010 | 1.6 | 0.003167133 | HSD17B10 |
| ENSSSCT00000025804 | 2.176 | 4.98E-05 | MCT13 |
| ENSSSCT00000022593 | 1.552 | 0.003913408 | IFI35 |
| ENSSSCT00000027087 | 1.545 | 0.002839738 | MCT11 |
| ENSSSCT00000019556 | 1.635 | 0.002966894 | LSMD1 |
| ENSSSCT00000019508 | 1.898 | 0.001411432 | MCT11 |
| ENSSSCT00000015328 | 1.797 | 0.000858363 | NHP2 |
| ENSSSCT00000017859 | 1.686 | 0.000741987 | AGXT |
| ENSSSCT00000017011 | 2.119 | 0.000133724 | G0s2 |
| ENSSSCT00000033526 | 2.022 | 0.000197181 | G0s2 |
| ENSSSCT00000010818 | 1.702 | 0.000891816 | MGC159817 |
| ENSSSCT00000031506 | 1.63 | 0.001737052 | UQCR10 |
| ENSSSCT00000029697 | 1.569 | 0.002267572 | hyi |
| ENSSSCT00000014098 | 1.5 | 0.00729018 | Doc2g |
| ENSSSCT00000019511 | 1.632 | 0.001752421 | C17orf49 |
| ENSSSCT00000014099 | 1.549 | 0.003406732 | NDUFV1 |
| ENSSSCT00000032779 | 1.885 | 0.001398943 | LST1 |
| ENSSSCT00000007791 | 1.65 | 0.004707708 | NXT1 |
| ENSSSCT00000028618 | 2.016 | 7.70E-05 | SPSB2 |
| ENSSSCT00000014243 | 1.919 | 0.000380998 | PRDX5 |
| ENSSSCT00000014979 | 2.004 | 2.49E-05 | MUC16 |
| ENSSSCT00000026071 | 1.824 | 0.000517054 | C9orf16 |
| ENSSSCT00000014187 | 1.671 | 0.000917695 | Rnaseh2c |
| ENSSSCT00000006206 | 1.725 | 0.001003223 | C9ORF119 |
| ENSSSCT00000014680 | 1.712 | 0.001200201 | Uqcr11 |
| ENSSSCT00000008694 | 1.59 | 0.001758517 | ROGDI |
| ENSSSCT00000006210 | 1.624 | 0.001026052 | SLC27A4 |
| ENSSSCT00000027115 | 1.775 | 0.001025035 | COPE |
| ENSSSCT00000003090 | 1.537 | 0.004194651 | TMEM208 |
| ENSSSCT00000000207 | 1.665 | 0.001462026 | TUBA1C |
| ENSSSCT00000006224 | 1.61 | 0.002483578 | ENDOG |
| ENSSSCT00000010999 | 3.159 | 3.71E-06 | IGLL1 |
| ENSSSCT00000011005 | 3.122 | 2.98E-06 | IGLL1 |
| ENSSSCT00000018217 | 1.584 | 0.003916406 | Rplp1 |
| ENSSSCT00000032603 | 1.731 | 0.000383439 | RPL21 |
| ENSSSCT00000013363 | 2.585 | 0.000104161 | RPL21 |
| ENSSSCT00000026810 | 1.574 | 0.002762073 | GSTK1 |
| ENSSSCT00000017841 | 1.587 | 0.001560072 | CHCHD2 |
| ENSSSCT00000018593 | 1.6 | 0.004639379 | ISCA1 |
| ENSSSCT00000026919 | 3.3 | 1.58E-09 | SULT1A1 |
| ENSSSCT00000029879 | 1.532 | 0.008930029 | TRAF4 |
| ENSSSCT00000032556 | 2.076 | 0.00023017 | PPIB |
| ENSSSCT00000027983 | 1.531 | 0.004839856 | VARS |
| ENSSSCT00000012010 | 1.516 | 0.004830247 | RPP25 |
| ENSSSCT00000024511 | 1.659 | 0.001525792 | RNH1 |
| ENSSSCT00000010941 | 1.646 | 0.004358387 | ZMAT5 |
| ENSSSCT00000031825 | 2.068 | 0.000112814 | Sigirr |
| ENSSSCT00000031762 | 1.995 | 0.000315323 | MAPK12 |
| ENSSSCT00000032257 | 1.542 | 0.004845739 | TTC27 |
| ENSSSCT00000023500 | 1.597 | 0.002219637 | PSMB1 |
| ENSSSCT00000030820 | 1.563 | 0.003043118 | ATP5J |
| ENSSSCT00000031542 | 2.634 | 0.00378148 | MT1A |
| ENSSSCT00000029094 | 2.054 | 0.002847409 | MT1A |
| ENSSSCT00000022297 | 1.68 | 0.002237631 | TDH |
| ENSSSCT00000023858 | 1.54 | 0.004678313 | EFNA4 |
| ENSSSCT00000029761 | 2.099 | 0.000306939 | Igh-VJ558 |
| ENSSSCT00000029668 | 1.863 | 0.000202302 | PACSIN3 |
| ENSSSCT00000003211 | 1.653 | 0.00401639 | ETV2 |
| ENSSSCT00000026250 | 1.504 | 0.003583438 | RUVBL1 |
| ENSSSCT00000008834 | 1.713 | 0.001553212 | TCEB2 |
| ENSSSCT00000024050 | 2.674 | 2.65E-05 | CKB |
| ENSSSCT00000026475 | 1.541 | 0.002762678 | TMEM120A |
| ENSSSCT00000012612 | 1.588 | 0.002277642 | CHL1 |
| ENSSSCT00000024134 | 1.532 | 0.002217346 | PHYH |
| ENSSSCT00000011059 | 1.732 | 0.001184957 | Thap7 |
| ENSSSCT00000002293 | 1.544 | 0.00347402 | DAD1 |
| ENSSSCT00000003212 | 1.649 | 0.001524158 | COX6B |
| ENSSSCT00000030728 | 1.78 | 0.000920538 | CHMP2A |
| ENSSSCT00000030677 | 1.675 | 0.000869678 | AKR1A1 |
| ENSSSCT00000002911 | 1.525 | 0.003421548 | GSTA2 |
| ENSSSCT00000016420 | 1.572 | 0.009547396 | NNMT |
| ENSSSCT00000002910 | 1.604 | 0.001408983 | GSTA2 |
| ENSSSCT00000015890 | 1.542 | 0.002328172 | FTH1 |
| ENSSSCT00000002913 | 2.077 | 0.000394794 | GCSH |
| ENSSSCT00000007843 | 1.599 | 0.007698418 | CPXM1 |
| ENSSSCT00000025816 | 1.746 | 0.003377943 | DLK1 |
| ENSSSCT00000031225 | 1.691 | 0.001330327 | DLK1 |
| ENSSSCT00000022661 | 1.847 | 0.002065264 | DLK1 |
| ENSSSCT00000026701 | 1.932 | 3.31E-05 | DLK1 |
| ENSSSCT00000024039 | 2.428 | 2.79E-05 | Samm50 |
| ENSSSCT00000035182 | 1.815 | 0.001354331 | PLP1 |
| ENSSSCT00000013705 | 2.106 | 0.000698803 | PLP1 |
| ENSSSCT00000034814 | 1.57 | 0.008873577 | PLP1 |
| ENSSSCT00000028582 | 2.763 | 2.02E-06 | GARNL3 |
| ENSSSCT00000006310 | 1.744 | 0.000649399 | GTF3C5 |
| ENSSSCT00000003046 | 1.573 | 0.00200971 | HP |
| ENSSSCT00000035383 | 1.605 | 0.001644747 | HP |
| ENSSSCT00000027856 | 6.472 | 5.21E-07 | PON3 |
| ENSSSCT00000029667 | 1.651 | 0.000981924 | ANAPC11 |
| ENSSSCT00000011452 | 1.889 | 0.000731199 | PPP1R3C |
| ENSSSCT00000026036 | 1.903 | 0.000197234 | PNKD |
| ENSSSCT00000027408 | 1.532 | 0.005647348 | GDPD1 |
| ENSSSCT00000000052 | 1.756 | 0.001181224 | NDUFA6 |
| ENSSSCT00000022955 | 1.582 | 0.001326234 | SH3BP2 |
| ENSSSCT00000032287 | 1.56 | 0.00339066 | EBP |
| ENSSSCT00000029368 | 1.98 | 6.02E-05 | HGD |
| ENSSSCT00000016435 | 1.535 | 0.003220911 | APOC3 |
| ENSSSCT00000026028 | 1.541 | 0.003525986 | AGPAT3 |
| ENSSSCT00000032543 | 1.569 | 0.003771068 | FCN2 |
| ENSSSCT00000026302 | 1.883 | 0.001846105 | TRPM8 |
| ENSSSCT00000027544 | 1.603 | 0.002252747 | BCS1L |
| ENSSSCT00000022692 | 1.64 | 0.001148468 | SPP2 |
| ENSSSCT00000010747 | 1.611 | 0.001067673 | HPD |
| ENSSSCT00000000067 | 1.555 | 0.004268273 | PMM1 |
| ENSSSCT00000031800 | 1.623 | 0.002002743 | ECHS1 |
| ENSSSCT00000003426 | 1.571 | 0.002293996 | APOE |
| ENSSSCT00000000399 | 1.547 | 0.004073765 | RPS26 |
| ENSSSCT00000024064 | 1.746 | 0.000677284 | CISD3 |
| ENSSSCT00000025319 | 1.735 | 0.001181885 | CISD3 |
| ENSSSCT00000023992 | 1.696 | 0.001601463 | DUS1L |
| ENSSSCT00000031269 | 1.547 | 0.003709999 | F12 |
| ENSSSCT00000031431 | 1.659 | 0.00091776 | AMT |
| ENSSSCT00000006407 | 1.801 | 0.000226861 | AGPAT2 |
| ENSSSCT00000005521 | 1.655 | 0.003055482 | RPL36AL |
| ENSSSCT00000003051 | 1.534 | 0.002965181 | NQO1 |
| ENSSSCT00000015476 | 1.653 | 0.001942364 | GLRX |
| ENSSSCT00000006411 | 1.565 | 0.003170204 | EDF1 |
| ENSSSCT00000018041 | 1.671 | 0.001170354 | Gm4332 |
| ENSSSCT00000001051 | 1.709 | 0.001296296 | MIOX |
| ENSSSCT00000024494 | 1.544 | 0.003412736 | UPP1 |
| ENSSSCT00000031649 | 2.337 | 5.55E-05 | PSMB3 |
| ENSSSCT00000003425 | 1.71 | 0.00069992 | APOC4 |
| ENSSSCT00000034762 | 1.513 | 0.009318243 | BST1 |
| ENSSSCT00000000229 | 1.755 | 0.000883468 | GPD1 |
| ENSSSCT00000004318 | 1.721 | 0.000929868 | UQCRH |
| ENSSSCT00000025654 | 1.585 | 0.004084465 | SIL1 |
| ENSSSCT00000026684 | 1.663 | 0.002303697 | DDIT3 |
| ENSSSCT00000023034 | 1.684 | 0.001854241 | LIPC |
| ENSSSCT00000004467 | 2.359 | 0.00105619 | SLC22A3 |
| ENSSSCT00000014248 | 1.71 | 0.001624102 | PHI-1 |
| ENSSSCT00000034776 | 2.033 | 0.009925182 | CLEC18A |
| ENSSSCT00000028632 | 1.924 | 0.000425275 | BATF |
| ENSSSCT00000014249 | 1.616 | 0.003605394 | Fkbp2 |
| ENSSSCT00000016157 | 1.5 | 0.005489972 | RGD1311634 |
| ENSSSCT00000026104 | 1.53 | 0.002830059 | MRPS15 |
| ENSSSCT00000019484 | 1.546 | 0.005994335 | ENO3 |
| ENSSSCT00000027826 | 1.629 | 0.002908188 | POLR1E |
| ENSSSCT00000028446 | 1.985 | 0.000328496 | ATOX1 |
| ENSSSCT00000002102 | 1.686 | 0.001091908 | SNUPN |
| ENSSSCT00000024096 | 1.693 | 0.000718026 | SEPW1 |
| ENSSSCT00000036269 | 1.516 | 0.007126782 | DOM3Z |
| ENSSSCT00000002929 | 1.605 | 0.003325735 | TUBB3 |
| ENSSSCT00000030699 | 1.547 | 0.004087332 | UROS |
| ENSSSCT00000023877 | 1.848 | 0.00055735 | C28H10ORF10 |
| ENSSSCT00000005257 | 1.541 | 0.006766208 | RHOV |
| ENSSSCT00000014464 | 1.683 | 0.000858356 | PACSIN3 |
| ENSSSCT00000003945 | 1.84 | 0.001363101 | PAQR7 |
| ENSSSCT00000014010 | 1.701 | 0.002019097 | FUNDC2 |
| ENSSSCT00000035762 | 1.513 | 0.006893988 | FUNDC2 |
| ENSSSCT00000011982 | 2.226 | 0.005390566 | FBP2 |
| ENSSSCT00000016380 | 2.275 | 0.000114489 | POU2AF1 |
| ENSSSCT00000009361 | 1.694 | 0.001381657 | KHK |
| ENSSSCT00000029365 | 1.553 | 0.006585783 | PPP1R14A |
| ENSSSCT00000011755 | 1.675 | 0.001302976 | BCCIP |
| ENSSSCT00000000417 | 1.712 | 0.000796382 | Myl6 |
| ENSSSCT00000000418 | 1.788 | 0.000405722 | MYL6 |
| ENSSSCT00000027267 | 1.844 | 0.000357762 | ACY1 |
| ENSSSCT00000026552 | 1.539 | 0.006629654 | TRMT1 |
| ENSSSCT00000005394 | 2.213 | 8.01E-05 | SERPINB10 |
| ENSSSCT00000018495 | 1.601 | 0.001981177 | BNIP1 |
| ENSSSCT00000012003 | 1.806 | 0.001462713 | CCL19 |
| ENSSSCT00000005924 | 1.681 | 0.001269708 | SEC61B |
| ENSSSCT00000014760 | 1.588 | 0.002358758 | LSDP5 |
| ENSSSCT00000011028 | 1.854 | 0.000478323 | MIF |
| ENSSSCT00000014771 | 1.701 | 0.001527128 | QIL1 |
| ENSSSCT00000004262 | 1.693 | 0.001569674 | C3H1orf123 |
| ENSSSCT00000016597 | 2.407 | 1.23E-05 | DCPS |
| ENSSSCT00000019487 | 1.537 | 0.003151085 | OMC |
| ENSSSCT00000030705 | 1.617 | 0.0030168 | TSSC1 |
| ENSSSCT00000018929 | 1.501 | 0.004988016 | RAMP2 |
| ENSSSCT00000027606 | 1.824 | 0.000434534 | PCBD2 |
| ENSSSCT00000029874 | 1.533 | 0.003229598 | ATPIF1 |
| ENSSSCT00000034316 | 1.818 | 0.000667126 | UBE1X |
| ENSSSCT00000009452 | 1.51 | 0.004967243 | IAH1 |
| ENSSSCT00000033137 | 1.539 | 0.006512905 | CSNK2B |
| ENSSSCT00000023908 | 1.74 | 0.001927872 | FARSB |
| ENSSSCT00000032393 | 1.539 | 0.00941028 | VpreB |
| ENSSSCT00000030327 | 1.614 | 0.005218634 | IL17D |
| ENSSSCT00000007144 | 1.55 | 0.002474053 | MTX1 |
| ENSSSCT00000004938 | 1.8 | 0.000191386 | HMGN3 |
| ENSSSCT00000000425 | 1.552 | 0.003338152 | CNPY2 |
| ENSSSCT00000013014 | 1.551 | 0.004056803 | COX17 |
| ENSSSCT00000006734 | 3.356 | 6.01E-05 | CA3 |
| ENSSSCT00000017649 | 1.738 | 0.001014459 | ZFAND2B |
| ENSSSCT00000029584 | 1.954 | 0.000226715 | TMEM111 |
| ENSSSCT00000015194 | 1.765 | 0.000615942 | TMEM111 |
| ENSSSCT00000027232 | 1.508 | 0.007700289 | FABP3 |
| ENSSSCT00000011768 | 1.947 | 0.000192425 | MGMT |
| ENSSSCT00000015974 | 1.622 | 0.003585472 | FXC1 |
| ENSSSCT00000007996 | 1.707 | 0.001707491 | ROMO1 |
| ENSSSCT00000004475 | 1.527 | 0.002922887 | MRPL18 |
| ENSSSCT00000000131 | 1.635 | 0.003349 | LGALS1 |
| ENSSSCT00000023998 | 1.695 | 0.001262732 | POLR2E |
| ENSSSCT00000032011 | 1.567 | 0.002321527 | LGALS3 |
| ENSSSCT00000000408 | 1.696 | 0.001170725 | Myl6 |
| ENSSSCT00000000409 | 1.742 | 0.000611558 | MYL6 |
| ENSSSCT00000032690 | 1.545 | 0.002754745 | VPS28 |
| ENSSSCT00000036001 | 1.571 | 0.008815254 | VPS28 |
| ENSSSCT00000029493 | 1.925 | 0.000517686 | SELM |
| ENSSSCT00000028302 | 1.505 | 0.004600194 | SAP18 |
| ENSSSCT00000003438 | 2.559 | 2.19E-05 | PGLYRP1 |
| ENSSSCT00000003652 | 1.647 | 0.001567842 | HSPBP1 |
| ENSSSCT00000032099 | 1.612 | 0.001980827 | ATP5J |
| ENSSSCT00000015336 | 1.59 | 0.002353274 | RGS14 |
| ENSSSCT00000000136 | 1.753 | 0.007059523 | CYTH4 |
| ENSSSCT00000016181 | 1.512 | 0.00572573 | MRPL48 |
| ENSSSCT00000019152 | 1.731 | 0.000730669 | NME1 |
| ENSSSCT00000033424 | 1.642 | 0.001542912 | APOM |
| ENSSSCT00000035639 | 1.546 | 0.003800166 | GK |
| ENSSSCT00000013347 | 1.698 | 0.000790623 | GK |
| ENSSSCT00000026325 | 1.519 | 0.005376997 | PSMD6 |
| ENSSSCT00000035872 | 1.671 | 0.002201458 | TRAF4 |
| ENSSSCT00000019347 | 1.741 | 0.001089551 | TRAF4 |
| ENSSSCT00000019150 | 1.759 | 0.001042728 | NME2 |
| ENSSSCT00000002252 | 1.521 | 0.002919569 | DHRS4 |
| ENSSSCT00000035335 | 1.718 | 0.001877576 | TSPAN6 |
| ENSSSCT00000005151 | 1.556 | 0.001976229 | SQRDL |
| ENSSSCT00000008361 | 1.547 | 0.002420713 | ATP5J2 |
| ENSSSCT00000008434 | 2.128 | 6.40E-05 | Hsp27 |
| ENSSSCT00000005626 | 1.525 | 0.003871155 | MRPS11 |
| ENSSSCT00000025225 | 1.84 | 0.000600198 | SIGIRR |
| ENSSSCT00000013469 | 1.507 | 0.003679997 | MAGED1 |
| ENSSSCT00000024555 | 1.667 | 0.001290208 | PHF23 |
| ENSSSCT00000008974 | 1.504 | 0.004614533 | Cox5b |
| ENSSSCT00000003231 | 1.608 | 0.002086288 | POLR2I |
| ENSSSCT00000008435 | 2.079 | 9.12E-05 | Hsp27 |
| ENSSSCT00000003530 | 1.576 | 0.001885185 | IRF3 |
| ENSSSCT00000008354 | 1.62 | 0.001380545 | ATP5J2 |
| ENSSSCT00000003573 | 1.941 | 0.000237117 | JOSD2 |
| ENSSSCT00000023610 | 1.748 | 0.000923181 | COX7A1 |
| ENSSSCT00000008363 | 1.787 | 0.001280878 | Bud31 |
| ENSSSCT00000017506 | 1.505 | 0.004337657 | HSPE1 |
| ENSSSCT00000018738 | 1.542 | 0.002947671 | HN1 |
| ENSSSCT00000013174 | 1.63 | 0.001955244 | ATP5O |
| ENSSSCT00000010177 | 1.652 | 0.002741525 | MIPEP |
| ENSSSCT00000007357 | 2.689 | 0.000577925 | HMGCS2 |
| ENSSSCT00000027911 | 1.804 | 0.000398977 | Rps9 |
| ENSSSCT00000025751 | 1.822 | 0.00088082 | PRG3 |
| ENSSSCT00000026731 | 1.634 | 0.00273417 | RAB20 |
| ENSSSCT00000006964 | 2.203 | 1.93E-05 | APOA2 |
| ENSSSCT00000000271 | 1.844 | 0.000285877 | KRT8 |
| ENSSSCT00000032085 | 1.666 | 0.003356577 | ACPT |
| ENSSSCT00000022312 | 1.682 | 0.001142725 | PHLDA2 |
| ENSSSCT00000027145 | 1.84 | 0.000455416 | IGKV@ |
| ENSSSCT00000030107 | 1.534 | 0.008655752 | IGKV@ |
| ENSSSCT00000035739 | 3.175 | 6.38E-09 | THY1 |
| ENSSSCT00000016488 | 3.635 | 6.56E-10 | THY1 |
| ENSSSCT00000030944 | 1.646 | 0.001170362 | Fam187b |
| ENSSSCT00000030152 | 1.899 | 0.000294371 | TMEM162 |
| ENSSSCT00000000272 | 2.335 | 2.97E-05 | KRT18 |
| ENSSSCT00000035196 | 1.558 | 0.008067405 | GADD45G |
| ENSSSCT00000035938 | 1.531 | 0.009534205 | GADD45G |
| ENSSSCT00000007086 | 1.79 | 0.000408275 | MRPL24 |
| ENSSSCT00000033746 | 1.719 | 0.000529326 | CLU |
| ENSSSCT00000036447 | 1.52 | 0.004982434 | S100A16 |
| ENSSSCT00000027420 | 2.415 | 0.001757094 | MT-III |
| ENSSSCT00000015222 | 2.048 | 0.000148333 | NDUFA13 |
| ENSSSCT00000003545 | 1.604 | 0.00209451 | ADM5 |
| ENSSSCT00000007211 | 1.544 | 0.00910035 | S100A14 |
| ENSSSCT00000013569 | 1.703 | 0.006592129 | CITED1 |
| ENSSSCT00000035046 | 1.845 | 0.000371908 | ETFB |
| ENSSSCT00000034479 | 1.618 | 0.001854208 | ETFB |
| ENSSSCT00000003588 | 1.935 | 0.000249239 | ETFB |
| ENSSSCT00000011788 | 1.686 | 0.001134381 | ZNF511 |
| ENSSSCT00000025040 | 1.786 | 0.000260103 | NAGS |
| ENSSSCT00000017135 | 1.683 | 0.000738714 | PTPN18 |
| ENSSSCT00000007217 | 2.897 | 9.98E-07 | S100A9 |
| ENSSSCT00000033945 | 2.417 | 6.31E-06 | S100A9 |
| ENSSSCT00000030564 | 3.258 | 6.57E-07 | LTF |
| ENSSSCT00000017767 | 2.01 | 0.000340062 | TRPM8 |
| ENSSSCT00000013313 | 1.52 | 0.005435407 | PRDX4 |
| ENSSSCT00000007218 | 3.301 | 5.60E-08 | S100A12 |
| ENSSSCT00000034229 | 1.759 | 7.63E-06 | S100A12 |
| ENSSSCT00000034956 | 1.706 | 0.001153626 | SIGLEC10 |
| ENSSSCT00000007219 | 2.714 | 1.06E-05 | S100A8 |
| ENSSSCT00000036517 | 2.318 | 6.00E-05 | S100A8 |
| ENSSSCT00000025655 | 1.768 | 0.000531475 | UBL4A |
| ENSSSCT00000006760 | 1.661 | 0.001056414 | NDUFB8 |
| ENSSSCT00000029520 | 1.82 | 0.000485061 | COX4I1 |
| ENSSSCT00000027845 | 1.678 | 0.001722276 | AKR1E2 |
| ENSSSCT00000014034 | 1.979 | 0.000353057 | polr2l |
| ENSSSCT00000035922 | 1.632 | 0.001907043 | RPS16 |
| ENSSSCT00000032834 | 1.571 | 0.002664743 | RPS16 |
| ENSSSCT00000035729 | 1.53 | 0.004182479 | RPS16 |
| ENSSSCT00000035513 | 1.547 | 0.003846425 | RPS16 |
| ENSSSCT00000017656 | 1.513 | 0.008199877 | Tuba4a |
| ENSSSCT00000028602 | 2.083 | 0.000260298 | NPG4 |
| ENSSSCT00000032419 | 2.484 | 2.63E-06 | NPG3 |
| ENSSSCT00000000139 | 1.787 | 0.000327385 | TSTD1 |
| ENSSSCT00000025426 | 1.534 | 0.003076148 | NFKBIB |
| ENSSSCT00000019495 | 1.56 | 0.002198155 | PSMB6 |
| ENSSSCT00000006488 | 1.574 | 0.005155555 | POPAR |
| ENSSSCT00000035252 | 1.606 | 0.00583948 | TH1L |
| ENSSSCT00000030874 | 3.392 | 4.94E-06 | NPG1 |
| ENSSSCT00000035688 | 1.687 | 0.00159348 | CRABP1 |
| ENSSSCT00000026082 | 1.515 | 0.004313036 | LAGE3 |
| ENSSSCT00000011807 | 1.761 | 0.000858138 | C10orf125 |
| ENSSSCT00000024097 | 1.688 | 0.002235915 | FSCN2 |
| ENSSSCT00000011811 | 1.766 | 0.000545338 | NDUFAB1 |
| ENSSSCT00000023695 | 1.628 | 0.0017429 | CRABP1 |
| ENSSSCT00000008249 | 1.536 | 0.002620046 | CTSZ |
| ENSSSCT00000032971 | 1.623 | 0.004798873 | BAT2 |
| ENSSSCT00000029011 | 1.783 | 0.000960342 | ROM1 |
| ENSSSCT00000015020 | 1.506 | 0.008276998 | CALR |
| ENSSSCT00000010819 | 1.966 | 0.000963309 | SDS |
| ENSSSCT00000024372 | 2.195 | 0.000522284 | SDS |
| ENSSSCT00000006146 | 1.557 | 0.003217429 | PSMB7 |
| ENSSSCT00000017657 | 2.027 | 0.000182741 | TUBA1A |
| ENSSSCT00000003716 | 1.647 | 0.001765461 | MRPL20 |
| ENSSSCT00000027323 | 1.896 | 0.000387176 | PMAP-23 |
| ENSSSCT00000011046 | 2.129 | 0.007548684 | SDF2L1 |
| ENSSSCT00000013976 | 1.552 | 0.004245502 | SSR4 |
| ENSSSCT00000033750 | 1.753 | 0.001767807 | SSR4 |
| ENSSSCT00000033661 | 1.601 | 0.006093212 | SSR4 |
| ENSSSCT00000012426 | 1.843 | 0.001366116 | PG-2 |
| ENSSSCT00000023318 | 1.92 | 0.000501357 | NDUFS7 |
| ENSSSCT00000031245 | 1.932 | 0.000488039 | NDUFS7 |
| ENSSSCT00000035142 | 1.657 | 0.002005237 | HSD17B10 |
| ENSSSCT00000022430 | 2.013 | 0.000361235 | DTNB |
| ENSSSCT00000006168 | 3.137 | 6.74E-08 | GARNL3 |
| ENSSSCT00000006169 | 1.737 | 0.007126219 | SLC2A8 |
| ENSSSCT00000018527 | 1.522 | 0.003848196 | ARGRS |
| ENSSSCT00000004155 | 1.569 | 0.007305585 | PDZK1IP1 |
| ENSSSCT00000017768 | 1.534 | 0.002532589 | SPP2 |
| ENSSSCT00000000447 | 1.56 | 0.001674062 | MYO1A |
| ENSSSCT00000029383 | 1.729 | 0.002223874 | C5H12ORF57 |
| ENSSSCT00000008995 | 3.176 | 3.61E-07 | FABP1 |
| ENSSSCT00000008081 | 1.667 | 0.001112483 | TOMM34 |
| ENSSSCT00000035108 | 1.675 | 0.001439565 | NDUFB9 |
| ENSSSCT00000006559 | 1.737 | 0.000880539 | NDUFB9 |
| ENSSSCT00000014614 | 1.693 | 0.007046808 | NUCB2 |
| ENSSSCT00000027640 | 1.55 | 0.004073837 | CLDN3 |
| ENSSSCT00000001678 | 1.714 | 0.000947308 | PFDN6 |
| ENSSSCT00000032296 | 1.534 | 0.003093227 | ESPN |
| ENSSSCT00000033656 | 2.226 | 2.14E-05 | CLDN4 |
| ENSSSCT00000008461 | 1.65 | 0.002082178 | CLDN4 |
| ENSSSCT00000019098 | 2.319 | 3.57E-05 | ATP5G1 |
| ENSSSCT00000029056 | 1.852 | 0.003792228 | SCNN1B |
| ENSSSCT00000006186 | 1.611 | 0.001640061 | FPGS |
| ENSSSCT00000007157 | 1.516 | 0.003927364 | PMVK |
| ENSSSCT00000002282 | 1.649 | 0.001287455 | MRPL52 |
| ENSSSCT00000007838 | 1.689 | 0.000744047 | MRPS26 |
| ENSSSCT00000003744 | 1.556 | 0.003825637 | ESPN |
| ENSSSCT00000027499 | 1.922 | 9.98E-05 | IGKV@ |
| ENSSSCT00000032222 | 1.609 | 0.002897553 | IGKV@ |
| ENSSSCT00000030671 | 2.283 | 2.18E-05 | IGKV@ |
| ENSSSCT00000034262 | 2.093 | 3.80E-05 | GCSH |
| ENSSSCT00000016370 | 1.549 | 0.009040498 | RPS12 |
| ENSSSCT00000026985 | 1.98 | 0.000465744 | DCXR |
| ENSSSCT00000022314 | 2.037 | 0.000108037 | GCSH |
| ENSSSCT00000023784 | 2.119 | 3.18E-05 | GCSH |
| ENSSSCT00000012772 | 1.746 | 0.001661908 | RPLP1 |
| ENSSSCT00000031370 | 1.516 | 0.006516169 | GCSH |
| ENSSSCT00000018610 | 1.599 | 0.003438435 | GPX3 |
| ENSSSCT00000029947 | 1.563 | 0.00569482 | hypothetical LOC100063160 |
| ENSSSCT00000007118 | 1.867 | 0.00025568 | mitogen-activated protein-binding protein-interacting protein-like |
| ENSSSCT00000022252 | 1.565 | 0.00262085 | similar to Alpha-1-antitrypsin precursor (Alpha-1 protease inhibitor) (Alpha-1-antiproteinase) |
| ENSSSCT00000017392 | 1.633 | 0.001570265 | ATP synthase lipid-binding protein, mitochondrial-like |
| ENSSSCT00000025887 | 1.703 | 0.000975909 | hypothetical protein LOC100458281 |
| ENSSSCT00000022688 | 1.618 | 0.003503729 | similar to multiple myeloma overexpression gene 2 |
| ENSSSCT00000023024 | 2.218 | 3.57E-05 | similar to H2A histone family, member O |
| ENSSSCT00000033053 | 1.697 | 0.001141456 | adenine nucleotide translocator 2 |
| ENSSSCT00000015043 | 1.83 | 0.001035875 | uncharacterized protein LOC113230-like |
| ENSSSCT00000008710 | 1.53 | 0.004428725 | hypothetical protein LOC100513918 |
| ENSSSCT00000002761 | 1.792 | 0.000219132 | alpha-1-antichymotrypsin 2 |
| ENSSSCT00000001093 | 1.591 | 0.007809652 | hypothetical protein LOC722947 |
| ENSSSCT00000024163 | 1.586 | 0.001902428 | similar to LOC124446 protein |
| ENSSSCT00000025484 | 1.733 | 0.00116079 | mediator of RNA polymerase II transcription subunit 29-like |
| ENSSSCT00000007310 | 1.65 | 0.001613832 | similar to H2A histone family, member O |
| ENSSSCT00000007747 | 1.569 | 0.00557458 | similar to CG8067-PA |
| ENSSSCT00000013422 | 1.785 | 0.00068432 | NADH dehydrogenase [ubiquinone] 1 beta subcomplex subunit 11, mitochondrial-like |
| ENSSSCT00000035719 | 1.855 | 0.000283704 | NADH dehydrogenase [ubiquinone] 1 beta subcomplex subunit 11, mitochondrial-like |
| ENSSSCT00000036490 | 1.742 | 0.000946513 | NADH dehydrogenase [ubiquinone] 1 beta subcomplex subunit 11, mitochondrial-like |
| ENSSSCT00000011516 | 1.722 | 0.001517622 | vasopressin-induced protein, 32kDa |
| ENSSSCT00000018632 | 1.615 | 0.002043027 | NADH dehydrogenase [ubiquinone] iron-sulfur protein 6, mitochondrial-like |
| ENSSSCT00000030384 | 1.547 | 0.009637333 | plasma cell-induced resident endoplasmic reticulum protein-like |
| ENSSSCT00000034774 | 1.579 | 0.004031626 | mCG146274-like |
| ENSSSCT00000035359 | 1.604 | 0.002775944 | hypothetical protein LOC737451 |
| ENSSSCT00000008544 | 1.713 | 0.001885933 | hypothetical protein LOC100065556 |
| ENSSSCT00000018637 | 1.562 | 0.002665864 | hypothetical protein LOC100520811 |
| ENSSSCT00000023371 | 1.579 | 0.00242475 | hypothetical protein LOC741013 |
| ENSSSCT00000028876 | 1.722 | 0.001009849 | ADP-sugar pyrophosphatase-like |
| ENSSSCT00000012162 | 1.649 | 0.003026959 | ADP-sugar pyrophosphatase-like |
| ENSSSCT00000013185 | 1.639 | 0.002961817 | carbonyl reductase [NADPH] 3-like |
| ENSSSCT00000008767 | 1.698 | 0.002581461 | hypothetical protein LOC100524737 |
| ENSSSCT00000014661 | 1.528 | 0.001011553 | similar to elastase 2, neutrophil preproprotein |
| ENSSSCT00000011711 | 1.593 | 0.005166397 | similar to glia maturation factor homologous protein |
| ENSSSCT00000025122 | 1.564 | 0.002581012 | hypothetical protein LOC100060661 |
| ENSSSCT00000012428 | 1.506 | 0.003851225 | hypothetical LOC100063824 |
| ENSSSCT00000002243 | 1.547 | 0.003472428 | similar to Hypothetical UPF0172 protein CG3501 |
| ENSSSCT00000019509 | 2.983 | 3.36E-06 | monocarboxylate transporter 13-like |
| ENSSSCT00000031562 | 1.91 | 1.65E-05 | antileukoproteinase-like |
| ENSSSCT00000003747 | 1.576 | 0.009303076 | similar to acyl-CoA hydrolase |
| ENSSSCT00000009095 | 1.501 | 0.004057684 | protease-associated domain-containing protein of 21 kDa-like |
| ENSSSCT00000015612 | 1.696 | 0.001039746 | cytochrome b-c1 complex subunit 8-like |
| ENSSSCT00000014100 | 2.124 | 0.000706939 | glutathione S-transferase P-like |
| ENSSSCT00000034850 | 1.87 | 0.001578828 | hypothetical protein LOC100455751 |
| ENSSSCT00000035276 | 1.751 | 0.000903145 | similar to Ig lambda chain V region 4A precursor |
| ENSSSCT00000034375 | 1.861 | 0.001527029 | similar to Ig lambda chain V region 4A precursor |
| ENSSSCT00000008984 | 2.151 | 2.73E-05 | region containing similar to NGF-binding Ig light chain; similar to IG KAPPA CHAIN V-V REGION K2 PRECURSOR |
| ENSSSCT00000013637 | 1.559 | 0.00541697 | COX assembly mitochondrial protein homolog |
| ENSSSCT00000034061 | 1.514 | 0.009170653 | BOLA class I histocompatibility antigen, alpha chain BL3-7-like |
| ENSSSCT00000036431 | 1.646 | 0.003418454 | similar to ARD1 N-acetyl transferase homologue |
| ENSSSCT00000032590 | 1.615 | 0.00546428 | similar to ARD1 N-acetyl transferase homologue |
| ENSSSCT00000036346 | 1.684 | 0.001903881 | similar to ARD1 N-acetyl transferase homologue |
| ENSSSCT00000034005 | 1.536 | 0.005041494 | similar to ARD1 N-acetyl transferase homologue |
| ENSSSCT00000032804 | 1.526 | 0.004597304 | alpha-2-macroglobulin |
| ENSSSCT00000032877 | 1.964 | 0.000762709 | B-cell receptor-associated protein 31 |
| ENSSSCT00000033403 | 2.182 | 0.000108346 | Chromosome 17: 66,541,057-66,545,114 |
| ENSSSCT00000033016 | 2.267 | 1.89E-05 | APCS |

**Down-regulated transcripts in liver**

| **Transcripts ID** | **Ratio** | **P Value** | **Transcripts** |
| --- | --- | --- | --- |
| ENSSSCT00000022484 | 0.587 | 0.0001701 | TOPAZ1 |
| ENSSSCT00000023848 | 0.45 | 0.0026172 | PTPD |
| ENSSSCT00000011647 | 0.419 | 0.0049669 | CCDC186 |
| ENSSSCT00000012626 | 0.268 | 4.04E-05 | EDEM1 |
| ENSSSCT00000009805 | 0.418 | 0.0021787 | AFM |
| ENSSSCT00000001299 | 0.385 | 0.0008189 | ZNF192 |
| ENSSSCT00000019236 | 0.319 | 0.0009362 | MED13 |
| ENSSSCT00000024721 | 0.309 | 0.0001538 | MED13 |
| ENSSSCT00000026163 | 0.461 | 0.0019951 | KIAA0528 |
| ENSSSCT00000017281 | 0.584 | 4.88E-05 | KCNJ3 |
| ENSSSCT00000005727 | 0.405 | 0.0070198 | PTPRD |
| ENSSSCT00000036304 | 0.615 | 0.003967 | TNFRSF11A |
| ENSSSCT00000005404 | 0.559 | 0.0008581 | TNFRSF11A |
| ENSSSCT00000005405 | 0.448 | 0.0076909 | KIAA1468 |
| ENSSSCT00000009845 | 0.435 | 0.0094167 | CCNG2 |
| ENSSSCT00000023810 | 0.445 | 0.0082825 | LCT |
| ENSSSCT00000016327 | 0.503 | 0.0033038 | SESN3 |
| ENSSSCT00000023093 | 0.577 | 0.0086259 | PTPN14 |
| ENSSSCT00000013170 | 0.47 | 0.0036321 | C21orf62 |
| ENSSSCT00000016975 | 0.374 | 0.0003329 | PTPN14 |
| ENSSSCT00000006946 | 0.469 | 0.0045834 | DDR2 |
| ENSSSCT00000024449 | 0.43 | 0.0035213 | NOTCH2 |
| ENSSSCT00000017550 | 0.285 | 0.0005942 | RAPH1 |
| ENSSSCT00000018367 | 0.455 | 0.006187 | C5orf51 |
| ENSSSCT00000025566 | 0.52 | 0.0034344 | LRBA |
| ENSSSCT00000028846 | 0.518 | 0.0053132 | ERCC2 |
| ENSSSCT00000029123 | 0.609 | 0.0020747 | MLL3 |
| ENSSSCT00000025866 | 0.238 | 1.26E-05 | ALR |
| ENSSSCT00000026487 | 0.405 | 0.0068589 | ALR |
| ENSSSCT00000004080 | 0.408 | 0.0056786 | ANKRD12 |
| ENSSSCT00000024669 | 0.42 | 0.0014335 | TP63 |
| ENSSSCT00000025253 | 0.501 | 0.008923 | KCND2 |
| ENSSSCT00000016725 | 0.43 | 0.0067399 | TMEM106B |
| ENSSSCT00000029379 | 0.533 | 0.0022795 | SMC5 |
| ENSSSCT00000029337 | 0.308 | 8.10E-05 | KLF9 |
| ENSSSCT00000015628 | 0.408 | 0.0016763 | Sec24a |
| ENSSSCT00000028090 | 0.29 | 2.60E-05 | KLB |
| ENSSSCT00000018666 | 0.348 | 0.0014265 | RNF213 |
| ENSSSCT00000018668 | 0.273 | 0.0009345 | RNF213 |
| ENSSSCT00000025780 | 0.539 | 0.0041313 | BRCA2 |
| ENSSSCT00000009956 | 0.407 | 0.0014632 | Kiaa1109 |
| ENSSSCT00000012826 | 0.532 | 0.0096624 | P2RY13 |
| ENSSSCT00000003009 | 0.413 | 0.0029986 | RFWD3 |
| ENSSSCT00000028204 | 0.537 | 0.0060487 | ZMIZ1 |
| ENSSSCT00000029245 | 0.529 | 0.0098843 | ZMIZ1 |
| ENSSSCT00000009817 | 0.445 | 0.0006758 | epiregulin |
| ENSSSCT00000030028 | 0.469 | 0.0041103 | CEP110 |
| ENSSSCT00000004784 | 0.405 | 0.0037465 | MDN1 |
| ENSSSCT00000009757 | 0.458 | 0.0098343 | CLOCK |
| ENSSSCT00000022817 | 0.534 | 0.003957 | SERINC5 |
| ENSSSCT00000015638 | 0.429 | 4.27E-05 | Fbxl21 |
| ENSSSCT00000025154 | 0.622 | 0.006686 | BRIP1 |
| ENSSSCT00000004793 | 0.404 | 0.0071492 | MANEA |
| ENSSSCT00000033279 | 0.421 | 0.0027835 | DLG1 |
| ENSSSCT00000025688 | 0.476 | 0.0099149 | ZNF814 |
| ENSSSCT00000022277 | 0.428 | 0.0070388 | ATPase |
| ENSSSCT00000004903 | 0.458 | 0.0098997 | TSPYL1 |
| ENSSSCT00000025281 | 0.427 | 0.0007275 | ZNF33B |
| ENSSSCT00000026345 | 0.503 | 0.0042783 | RNF213 |
| ENSSSCT00000000914 | 0.457 | 0.0088347 | KIAA1033 |
| ENSSSCT00000032996 | 0.525 | 0.0034122 | PTK2 |
| ENSSSCT00000029251 | 0.511 | 0.0055091 | PTK2 |
| ENSSSCT00000024349 | 0.504 | 0.0007441 | SPG11 |
| ENSSSCT00000026935 | 0.629 | 0.0051195 | GalR1 |
| ENSSSCT00000013138 | 0.448 | 0.0081214 | STCH |
| ENSSSCT00000031109 | 0.352 | 0.0006439 | CGNL1 |
| ENSSSCT00000027714 | 0.34 | 0.001188 | TP53INP1 |
| ENSSSCT00000029217 | 0.41 | 0.0016167 | TRIM5 |
| ENSSSCT00000030687 | 0.469 | 0.0006732 | UTRN |
| ENSSSCT00000016643 | 0.265 | 0.0010576 | ZBED6 |
| ENSSSCT00000022585 | 0.504 | 0.0041196 | TMPRSS11D |
| ENSSSCT00000036604 | 0.432 | 0.0054238 | ATRX |
| ENSSSCT00000012955 | 0.399 | 0.0032316 | PAK2 |
| ENSSSCT00000027203 | 0.337 | 0.0035174 | LRP1 |
| ENSSSCT00000023139 | 0.434 | 0.0019821 | HERC2 |
| ENSSSCT00000026115 | 0.536 | 0.0012655 | PHACTR2 |
| ENSSSCT00000034643 | 0.44 | 0.0047808 | ERAP1 |
| ENSSSCT00000024219 | 0.527 | 0.0077626 | PRRC1 |
| ENSSSCT00000013871 | 0.605 | 0.005367 | ZNF449 |
| ENSSSCT00000000547 | 0.418 | 0.0051767 | ZFC3H1 |
| ENSSSCT00000031721 | 0.594 | 0.0032986 | SLC23A2 |
| ENSSSCT00000009102 | 0.306 | 6.71E-06 | CYP26B1 |
| ENSSSCT00000000548 | 0.323 | 0.000964 | ZFC3H1 |
| ENSSSCT00000018496 | 0.402 | 0.0034538 | C5orf41 |
| ENSSSCT00000028955 | 0.428 | 0.0096868 | SERINC1 |
| ENSSSCT00000025072 | 0.271 | 0.0001067 | EAR-1r |
| ENSSSCT00000005104 | 0.513 | 0.0041751 | MYO5C |
| ENSSSCT00000013455 | 0.541 | 0.0047153 | ubusp22-a |
| ENSSSCT00000016778 | 0.36 | 0.0033319 | HGF |
| ENSSSCT00000027624 | 0.49 | 0.0034995 | ATP8B2 |
| ENSSSCT00000023953 | 0.383 | 0.0004019 | ERO1B |
| ENSSSCT00000012754 | 0.48 | 0.0050525 | STAG1 |
| ENSSSCT00000005726 | 0.37 | 0.006653 | PTPRD |
| ENSSSCT00000029124 | 0.409 | 0.0022235 | PTPRD |
| ENSSSCT00000026060 | 0.619 | 0.0031836 | AQR |
| ENSSSCT00000001932 | 0.431 | 0.0047341 | CD2AP |
| ENSSSCT00000009429 | 0.426 | 0.0017155 | MYCN |
| ENSSSCT00000002683 | 0.338 | 0.0003496 | SEL1L |
| ENSSSCT00000007658 | 0.49 | 0.0099321 | PCM-1 |
| ENSSSCT00000022881 | 0.465 | 0.009844 | MAP2K2 |
| ENSSSCT00000005415 | 0.427 | 0.0027263 | LMAN1L |
| ENSSSCT00000011239 | 0.421 | 0.0074371 | Eif4ebp2 |
| ENSSSCT00000019463 | 0.382 | 0.0007128 | FBXO39 |
| ENSSSCT00000008003 | 0.469 | 0.0088292 | EPB41L3 |
| ENSSSCT00000025669 | 0.572 | 0.0073278 | SPAG9 |
| ENSSSCT00000002395 | 0.56 | 0.0038752 | TEP1 |
| ENSSSCT00000012115 | 0.374 | 0.0041554 | THNSL1 |
| ENSSSCT00000013223 | 0.418 | 0.0009837 | ADARB1 |
| ENSSSCT00000005149 | 0.457 | 0.0069765 | SEMA6D |
| ENSSSCT00000024799 | 0.507 | 0.0057818 | SEMA6D |
| ENSSSCT00000034959 | 0.577 | 0.0026063 | BAT3 |
| ENSSSCT00000016605 | 0.447 | 0.0047852 | ARHGAP32 |
| ENSSSCT00000016789 | 0.461 | 0.009078 | FAM185A |
| ENSSSCT00000002526 | 0.49 | 0.0064153 | AKAP5 |
| ENSSSCT00000000877 | 0.347 | 0.0008745 | Snat2 |
| ENSSSCT00000029107 | 0.533 | 0.0046366 | Slc9a8 |
| ENSSSCT00000024610 | 0.397 | 0.0056532 | EP400 |
| ENSSSCT00000027939 | 0.425 | 0.0084353 | EP400 |
| ENSSSCT00000015573 | 0.334 | 0.0004733 | PRRC1 |
| ENSSSCT00000006805 | 0.397 | 0.0026478 | VCPIP1 |
| ENSSSCT00000014526 | 0.463 | 0.0001627 | EHF |
| ENSSSCT00000024429 | 0.488 | 0.0004634 | KIAA1217 |
| ENSSSCT00000028762 | 0.527 | 0.0092831 | KIAA2018 |
| ENSSSCT00000034180 | 0.522 | 0.0016351 | THO2 |
| ENSSSCT00000024635 | 0.459 | 0.0044483 | PNPT1 |
| ENSSSCT00000024292 | 0.385 | 0.0098135 | TJP1 |
| ENSSSCT00000012972 | 0.415 | 0.0072773 | HEG1 |
| ENSSSCT00000025511 | 0.423 | 0.0025573 | HEG1 |
| ENSSSCT00000004140 | 0.48 | 0.0039746 | ZNF271 |
| ENSSSCT00000002694 | 0.372 | 0.004863 | PTPN21 |
| ENSSSCT00000015459 | 0.44 | 0.0003592 | MBLAC2 |
| ENSSSCT00000002947 | 0.416 | 0.0082805 | ZCCHC14 |
| ENSSSCT00000012306 | 0.428 | 0.0039568 | TRANK1 |
| ENSSSCT00000008222 | 0.176 | 2.36E-05 | PCK1 |
| ENSSSCT00000032634 | 0.256 | 5.30E-05 | PCK1 |
| ENSSSCT00000034727 | 0.221 | 2.51E-06 | PCK1 |
| ENSSSCT00000034470 | 0.183 | 2.79E-05 | PCK1 |
| ENSSSCT00000033314 | 0.245 | 2.10E-05 | PCK1 |
| ENSSSCT00000010705 | 0.45 | 0.0061127 | ZNF664 |
| ENSSSCT00000012798 | 0.427 | 0.002733 | PLSCR4 |
| ENSSSCT00000026201 | 0.426 | 0.0091832 | CAMK1D |
| ENSSSCT00000034337 | 0.419 | 0.0027641 | Hao2 |
| ENSSSCT00000030778 | 0.505 | 0.0081386 | KIAA1797 |
| ENSSSCT00000011628 | 0.193 | 3.07E-05 | TECTB |
| ENSSSCT00000015375 | 0.46 | 0.0072891 | Gfm2 |
| ENSSSCT00000008383 | 0.405 | 0.003809 | Zkscan1 |
| ENSSSCT00000014442 | 0.376 | 0.0025535 | FNBP4 |
| ENSSSCT00000015505 | 0.506 | 0.009078 | FER |
| ENSSSCT00000026299 | 0.358 | 0.0017365 | HSPG2 |
| ENSSSCT00000023415 | 0.453 | 0.0070141 | TOPBP1 |
| ENSSSCT00000033717 | 0.454 | 0.0074638 | SMC1A |
| ENSSSCT00000032848 | 0.37 | 0.0002384 | PER1 |
| ENSSSCT00000034988 | 0.421 | 3.11E-05 | PER1 |
| ENSSSCT00000019569 | 0.363 | 0.0001446 | PER1 |
| ENSSSCT00000017261 | 0.335 | 0.0023535 | HERC2 |
| ENSSSCT00000019362 | 0.453 | 0.0047661 | TAOK1 |
| ENSSSCT00000027995 | 0.437 | 0.0086903 | FNBP4 |
| ENSSSCT00000012879 | 0.451 | 0.004863 | CCDC39 |
| ENSSSCT00000012774 | 0.506 | 0.0053225 | SLC25A36 |
| ENSSSCT00000017793 | 0.449 | 0.0039738 | PER2 |
| ENSSSCT00000017688 | 0.493 | 0.008981 | COL4A4 |
| ENSSSCT00000033392 | 0.325 | 0.0021831 | MBNL3 |
| ENSSSCT00000034072 | 0.374 | 0.0038518 | Mbnl3 |
| ENSSSCT00000034918 | 0.461 | 0.0039427 | MBNL3 |
| ENSSSCT00000018526 | 0.358 | 0.0004092 | PANK3 |
| ENSSSCT00000029581 | 0.508 | 0.0044903 | PSME4 |
| ENSSSCT00000012560 | 0.427 | 0.0011166 | FAM107A |
| ENSSSCT00000033532 | 0.353 | 0.0007871 | UBR5 |
| ENSSSCT00000022974 | 0.414 | 7.44E-05 | UBR5 |
| ENSSSCT00000031893 | 0.402 | 0.0012649 | ARHGEF10 |
| ENSSSCT00000036362 | 0.639 | 0.0036362 | KIAA0556 |
| ENSSSCT00000011037 | 0.606 | 0.0021106 | ZNF70 |
| ENSSSCT00000014028 | 0.586 | 0.0063276 | PKA |
| ENSSSCT00000026945 | 0.32 | 2.25E-05 | NINL |
| ENSSSCT00000017478 | 0.506 | 0.0057914 | TMEM194B |
| ENSSSCT00000031953 | 0.458 | 0.0085985 | MLL2 |
| ENSSSCT00000028773 | 0.412 | 0.0083253 | TPCN1 |
| ENSSSCT00000024155 | 0.46 | 0.0081255 | KLHL15 |
| ENSSSCT00000033174 | 0.493 | 0.0051808 | BRWD3 |
| ENSSSCT00000031239 | 0.422 | 0.0017449 | BRWD3 |
| ENSSSCT00000028300 | 0.543 | 0.0078688 | PEG10 |
| ENSSSCT00000032473 | 0.399 | 0.0071789 | DLC1 |
| ENSSSCT00000027157 | 0.591 | 0.0029326 | ZNF148 |
| ENSSSCT00000018350 | 0.377 | 0.0024718 | LIFR |
| ENSSSCT00000018353 | 0.386 | 0.0046823 | RICTOR |
| ENSSSCT00000025457 | 0.206 | 3.80E-08 | SASS6 |
| ENSSSCT00000031488 | 0.443 | 0.008969 | PHIP |
| ENSSSCT00000028665 | 0.2 | 1.13E-05 | LEPR |
| ENSSSCT00000030540 | 0.611 | 0.0033944 | PLEKHA5 |
| ENSSSCT00000029338 | 0.466 | 0.006577 | GON4L |
| ENSSSCT00000024995 | 0.435 | 0.0050671 | Shank3 |
| ENSSSCT00000027335 | 0.397 | 0.0070442 | AOX1 |
| ENSSSCT00000002818 | 0.273 | 0.0007452 | DYNC1H1 |
| ENSSSCT00000022741 | 0.327 | 0.0002078 | DDX58 |
| ENSSSCT00000015388 | 0.352 | 0.0026784 | IQGAP2 |
| ENSSSCT00000029291 | 0.411 | 0.009018 | NNT |
| ENSSSCT00000030117 | 0.19 | 7.07E-05 | APOB |
| ENSSSCT00000009661 | 0.588 | 0.0086016 | FRYL |
| ENSSSCT00000022470 | 0.505 | 0.0003855 | PTCHD2 |
| ENSSSCT00000023233 | 0.377 | 0.0005462 | AK3L1 |
| ENSSSCT00000022649 | 0.298 | 8.78E-05 | NCOA2 |
| ENSSSCT00000027571 | 0.384 | 0.0073144 | EGFR |
| ENSSSCT00000030047 | 0.424 | 0.0024977 | PGM2 |
| ENSSSCT00000009679 | 0.415 | 0.0017488 | KIT |
| ENSSSCT00000027588 | 0.389 | 0.0022951 | ASH1L |
| ENSSSCT00000030747 | 0.516 | 0.0054139 | KLF11 |
| ENSSSCT00000031830 | 0.371 | 0.0052851 | CYP7B1 |
| ENSSSCT00000031325 | 0.638 | 0.0008559 | VPS13C |
| ENSSSCT00000023328 | 0.38 | 0.0004138 | MPV17L |
| ENSSSCT00000012616 | 0.497 | 0.0050145 | CNTN4 |
| ENSSSCT00000002707 | 0.517 | 0.0009926 | RPS6KA5 |
| ENSSSCT00000017988 | 0.351 | 0.002824 | CREB3L2 |
| ENSSSCT00000031984 | 0.464 | 0.000385 | ZBTB16 |
| ENSSSCT00000006821 | 0.285 | 0.0001744 | TTPA |
| ENSSSCT00000027968 | 0.416 | 0.0037028 | RNF123 |
| ENSSSCT00000012884 | 0.35 | 0.0002005 | ATP11B |
| ENSSSCT00000034458 | 0.402 | 0.0022262 | ITGA1 |
| ENSSSCT00000024626 | 0.498 | 0.0022227 | LPIN1 |
| ENSSSCT00000032020 | 0.667 | 0.0010319 | BRWD1 |
| ENSSSCT00000023238 | 0.423 | 0.0074658 | C3AR1 |
| ENSSSCT00000031714 | 0.484 | 0.0064091 | PTPRM |
| ENSSSCT00000013203 | 0.375 | 0.0008282 | Igsf5 |
| ENSSSCT00000033178 | 0.597 | 0.0004754 | GABRQ |
| ENSSSCT00000013945 | 0.377 | 1.62E-05 | GABRQ |
| ENSSSCT00000031247 | 0.418 | 0.0009352 | HOOK3 |
| ENSSSCT00000012502 | 0.546 | 0.0089015 | RAD54L2 |
| ENSSSCT00000025392 | 0.56 | 0.0057309 | TXNDC16 |
| ENSSSCT00000000671 | 0.408 | 0.0010578 | LRP6 |
| ENSSSCT00000029494 | 0.388 | 0.0008652 | HOOK3 |
| ENSSSCT00000014289 | 0.455 | 0.0088189 | FADS2 |
| ENSSSCT00000029912 | 0.417 | 0.0037601 | KIF13A |
| ENSSSCT00000011413 | 0.485 | 0.0051118 | DKK1 |
| ENSSSCT00000010234 | 0.403 | 0.0010843 | FRY |
| ENSSSCT00000027916 | 0.381 | 0.0011114 | SLC12A6 |
| ENSSSCT00000010233 | 0.661 | 0.004899 | FRY |
| ENSSSCT00000011650 | 0.445 | 0.0078006 | ABLIM1 |
| ENSSSCT00000011414 | 0.397 | 0.0002184 | PRKG1 |
| ENSSSCT00000000515 | 0.416 | 0.0041581 | HELB |
| ENSSSCT00000031962 | 0.483 | 0.0004176 | MAP4K3 |
| ENSSSCT00000010736 | 0.42 | 0.0076273 | MLXIP |
| ENSSSCT00000004213 | 0.634 | 0.0090449 | DNAJC6 |
| ENSSSCT00000030746 | 0.448 | 0.0093774 | ITPR1 |
| ENSSSCT00000004515 | 0.621 | 0.0060509 | ESR1 |
| ENSSSCT00000001012 | 0.423 | 0.003666 | TMTC3 |
| ENSSSCT00000018798 | 0.517 | 0.0084749 | Prkca |
| ENSSSCT00000006306 | 0.602 | 0.007512 | TSC1 |
| ENSSSCT00000015303 | 0.439 | 0.0054901 | MAPK9 |
| ENSSSCT00000027715 | 0.515 | 0.0099289 | PDS5B |
| ENSSSCT00000004214 | 0.374 | 0.0003011 | AK4 |
| ENSSSCT00000028712 | 0.379 | 0.0009524 | PARP4 |
| ENSSSCT00000009614 | 0.451 | 0.0055734 | RFC1 |
| ENSSSCT00000015739 | 0.444 | 0.0062837 | NR3C1 |
| ENSSSCT00000032298 | 0.605 | 0.0053458 | PDS5B |
| ENSSSCT00000011453 | 0.455 | 0.0093688 | TNKS2 |
| ENSSSCT00000032992 | 0.422 | 0.0070629 | JAK1 |
| ENSSSCT00000027790 | 0.626 | 0.0074673 | HERC1 |
| ENSSSCT00000013209 | 0.362 | 0.008018 | MX2 |
| ENSSSCT00000029309 | 0.461 | 0.0046178 | SMCHD1 |
| ENSSSCT00000034013 | 0.339 | 0.0021495 | IL6ST |
| ENSSSCT00000018422 | 0.346 | 0.0039525 | IL6ST |
| ENSSSCT00000028756 | 0.334 | 0.0001453 | EDEM1 |
| ENSSSCT00000024825 | 0.415 | 0.0034801 | HERPUD1 |
| ENSSSCT00000018421 | 0.56 | 0.0073484 | IL31RA |
| ENSSSCT00000012937 | 0.45 | 0.0065104 | ATP13A3 |
| ENSSSCT00000028020 | 0.535 | 5.14E-05 | BBS12 |
| ENSSSCT00000009616 | 0.546 | 0.0036049 | RFC1 |
| ENSSSCT00000018117 | 0.443 | 0.000554 | FOXP2 |
| ENSSSCT00000015415 | 0.307 | 2.05E-07 | JMY |
| ENSSSCT00000006788 | 0.301 | 0.0009409 | NCOA2 |
| ENSSSCT00000006789 | 0.306 | 0.0009725 | NCOA2 |
| ENSSSCT00000024545 | 0.451 | 0.0006574 | SMCHD1 |
| ENSSSCT00000005492 | 0.572 | 4.06E-05 | THSD4 |
| ENSSSCT00000029286 | 0.617 | 0.0075308 | SLC7A11 |
| ENSSSCT00000016707 | 0.218 | 8.97E-09 | PDK4 |
| ENSSSCT00000032203 | 0.508 | 0.0018734 | ATRNL1 |
| ENSSSCT00000029218 | 0.423 | 0.0076576 | DOCK4 |
| ENSSSCT00000018811 | 0.386 | 0.0010218 | ERN1 |
| ENSSSCT00000009619 | 0.43 | 0.0038166 | N4BP2 |
| ENSSSCT00000007372 | 0.406 | 0.0051153 | MAN1A2 |
| ENSSSCT00000030150 | 0.547 | 0.0077056 | KDM5A |
| ENSSSCT00000023353 | 0.421 | 0.0072694 | KDM5A |
| ENSSSCT00000030075 | 0.448 | 0.00092 | COG3 |
| ENSSSCT00000015509 | 0.403 | 0.0070611 | PJA2 |
| ENSSSCT00000025689 | 0.401 | 0.0006303 | ZNF295 |
| ENSSSCT00000028914 | 0.563 | 0.0062663 | HECW2 |
| ENSSSCT00000000570 | 0.428 | 0.0067674 | PKP2 |
| ENSSSCT00000010196 | 0.434 | 0.0068919 | LNX2 |
| ENSSSCT00000000819 | 0.385 | 0.0037758 | ERC1 |
| ENSSSCT00000023450 | 0.452 | 0.0046419 | ATF7 |
| ENSSSCT00000000299 | 0.459 | 0.0075834 | ATF7 |
| ENSSSCT00000023310 | 0.422 | 0.0015458 | VPS13C |
| ENSSSCT00000006817 | 0.383 | 0.0015831 | CYP7B1 |
| ENSSSCT00000009755 | 0.4 | 0.0015087 | CLOCK |
| ENSSSCT00000011456 | 0.374 | 0.0007509 | Cpeb3 |
| ENSSSCT00000022268 | 0.475 | 0.001811 | UBR1 |
| ENSSSCT00000024242 | 0.484 | 0.0086188 | REST |
| ENSSSCT00000009461 | 0.373 | 0.002928 | IRG6 |
| ENSSSCT00000032161 | 0.529 | 0.0091401 | PLEKHH2 |
| ENSSSCT00000029515 | 0.462 | 0.0035511 | NEO1 |
| ENSSSCT00000035614 | 0.322 | 0.003303 | SCD |
| ENSSSCT00000035292 | 0.354 | 0.0054695 | SCD |
| ENSSSCT00000035482 | 0.358 | 0.006142 | SCD |
| ENSSSCT00000030811 | 0.459 | 0.0079566 | FNDC3B |
| ENSSSCT00000031248 | 0.446 | 0.0074925 | FNDC3B |
| ENSSSCT00000005210 | 0.35 | 0.0004546 | TTBK2 |
| ENSSSCT00000016919 | 0.444 | 0.0097273 | CEP350 |
| ENSSSCT00000004785 | 0.45 | 0.0034445 | MDN1 |
| ENSSSCT00000004407 | 0.408 | 0.0038052 | MGC148601 |
| ENSSSCT00000004792 | 0.515 | 0.0022381 | EPHA7 |
| ENSSSCT00000031839 | 0.488 | 0.0003484 | FGD4 |
| ENSSSCT00000035696 | 0.542 | 0.0069981 | NBN |
| ENSSSCT00000032175 | 0.605 | 0.0061769 | LAMA2 |
| ENSSSCT00000030852 | 0.554 | 0.0067475 | LAMA2 |
| ENSSSCT00000027576 | 0.463 | 0.0085606 | DHX36 |
| ENSSSCT00000003052 | 0.316 | 0.000146 | NFAT5 |
| ENSSSCT00000015477 | 0.377 | 0.0010954 | ELL2 |
| ENSSSCT00000007570 | 0.549 | 0.0043614 | HFM1 |
| ENSSSCT00000031816 | 0.438 | 0.0067559 | PTPRS |
| ENSSSCT00000028903 | 0.572 | 0.0030594 | COL4A5 |
| ENSSSCT00000028101 | 0.605 | 0.0070843 | ADAM10 |
| ENSSSCT00000004807 | 0.42 | 0.0056706 | SFRS18 |
| ENSSSCT00000024435 | 0.465 | 0.0074406 | DENND5B |
| ENSSSCT00000003790 | 0.357 | 0.0001202 | PTCHD2 |
| ENSSSCT00000017958 | 0.43 | 0.0044518 | BRAF |
| ENSSSCT00000015517 | 0.372 | 0.0009551 | STARD4 |
| ENSSSCT00000010657 | 0.357 | 0.0005289 | DDX60 |
| ENSSSCT00000031450 | 0.32 | 7.65E-05 | KIAA1033 |
| ENSSSCT00000018585 | 0.414 | 0.0082039 | LARP1 |
| ENSSSCT00000001952 | 0.38 | 0.000699 | PKHD1 |
| ENSSSCT00000015889 | 0.452 | 0.0058048 | ZFP62 |
| ENSSSCT00000023196 | 0.519 | 0.0036668 | PGAP1 |
| ENSSSCT00000000911 | 0.407 | 0.0031721 | SLC41A2 |
| ENSSSCT00000016642 | 0.422 | 0.0015393 | ATP2B4 |
| ENSSSCT00000005236 | 0.379 | 0.0019268 | MGA |
| ENSSSCT00000018832 | 0.634 | 0.0061352 | TANC2 |
| ENSSSCT00000010718 | 0.406 | 0.0063547 | SBNO1 |
| ENSSSCT00000013874 | 0.577 | 0.008751 | ZNF449 |
| ENSSSCT00000026661 | 0.541 | 0.009818 | USP32 |
| ENSSSCT00000006731 | 0.58 | 0.0055049 | ATP6V0D2 |
| ENSSSCT00000017097 | 0.455 | 0.0074763 | CCNT2 |
| ENSSSCT00000009218 | 0.379 | 6.50E-05 | NRXN1 |
| ENSSSCT00000009219 | 0.579 | 0.0007327 | NRXN1 |
| ENSSSCT00000001825 | 0.391 | 0.0075136 | UBR2 |
| ENSSSCT00000017391 | 0.424 | 0.0034297 | ATF2 |
| ENSSSCT00000029113 | 0.447 | 0.0063364 | TRIM6-TRIM34 |
| ENSSSCT00000030872 | 0.516 | 0.0055212 | NETO2 |
| ENSSSCT00000000843 | 0.441 | 0.0053362 | KIF21A |
| ENSSSCT00000018491 | 0.322 | 0.001243 | CPEB4 |
| ENSSSCT00000008924 | 0.441 | 0.0045524 | FHL2 |
| ENSSSCT00000024808 | 0.541 | 0.0023888 | ATG10 |
| ENSSSCT00000002517 | 0.655 | 0.0087885 | AKAP13 |
| ENSSSCT00000031293 | 0.421 | 0.003448 | AKAP13 |
| ENSSSCT00000010687 | 0.333 | 0.0001101 | RIMBP2 |
| ENSSSCT00000013161 | 0.351 | 0.0018311 | BACH1 |
| ENSSSCT00000017740 | 0.429 | 0.0058854 | GIGYF2 |
| ENSSSCT00000002516 | 0.363 | 0.002856 | AKAP13 |
| ENSSSCT00000022914 | 0.458 | 0.0085252 | DICER1 |
| ENSSSCT00000010924 | 0.376 | 0.0015346 | TTC28 |
| ENSSSCT00000005700 | 0.38 | 0.0036652 | DENND4C |
| ENSSSCT00000025982 | 0.436 | 0.0037554 | FIGN |
| ENSSSCT00000009572 | 0.436 | 0.002655 | SLIT2 |
| ENSSSCT00000036206 | 0.549 | 0.0077636 | PRLR |
| ENSSSCT00000005538 | 0.425 | 0.0026417 | NIN |
| ENSSSCT00000032295 | 0.443 | 0.0055607 | NIN |
| ENSSSCT00000026076 | 0.535 | 0.0049864 | NCBP1 |
| ENSSSCT00000009277 | 0.457 | 0.0072183 | MAP4K3 |
| ENSSSCT00000028791 | 0.414 | 0.0059387 | USP34 |
| ENSSSCT00000009178 | 0.428 | 0.0085047 | USP34 |
| ENSSSCT00000034126 | 0.343 | 0.004519 | LRP1 |
| ENSSSCT00000000235 | 0.458 | 0.0073471 | LARP4 |
| ENSSSCT00000026142 | 0.422 | 0.0031693 | DAPK1 |
| ENSSSCT00000011293 | 0.439 | 0.0060228 | MYST4 |
| ENSSSCT00000016884 | 0.378 | 0.006227 | RC3H1 |
| ENSSSCT00000009833 | 0.504 | 0.0093717 | CXCL11 |
| ENSSSCT00000015016 | 0.427 | 0.003279 | NFIX |
| ENSSSCT00000007341 | 0.384 | 0.0016796 | FMO5 |
| ENSSSCT00000001166 | 0.461 | 0.0093582 | KIF13A |
| ENSSSCT00000023668 | 0.393 | 0.0006622 | KIF13A |
| ENSSSCT00000025839 | 0.361 | 0.0062106 | PUM1 |
| ENSSSCT00000008609 | 0.386 | 0.0012951 | ACSM2B |
| ENSSSCT00000010212 | 0.441 | 0.0039819 | FLT1 |
| ENSSSCT00000030187 | 0.543 | 0.0064254 | IL10RA |
| ENSSSCT00000031936 | 0.384 | 0.0012511 | MTMR9 |
| ENSSSCT00000009978 | 0.394 | 0.0006849 | SYNPO2 |
| ENSSSCT00000009979 | 0.383 | 0.0016809 | SEC24D |
| ENSSSCT00000008464 | 0.572 | 0.0062832 | Gtf2ird1 |
| ENSSSCT00000005922 | 0.597 | 0.0012823 | TGFBR1 |
| ENSSSCT00000009280 | 0.387 | 0.001854 | SOS1 |
| ENSSSCT00000025600 | 0.353 | 0.000307 | CLK1 |
| ENSSSCT00000024624 | 0.489 | 0.0042014 | TTC37 |
| ENSSSCT00000024141 | 0.63 | 0.0078938 | TTC37 |
| ENSSSCT00000024247 | 0.509 | 0.0088137 | EIF2AK4 |
| ENSSSCT00000005097 | 0.3 | 0.0002097 | ONECUT1 |
| ENSSSCT00000016777 | 0.423 | 0.0079924 | CACNA2D1 |
| ENSSSCT00000032520 | 0.466 | 0.008795 | SLA/LP |
| ENSSSCT00000003847 | 0.385 | 0.0036435 | RSC1A1 |
| ENSSSCT00000009281 | 0.31 | 0.0002259 | DHX57 |
| ENSSSCT00000030436 | 0.462 | 0.0040458 | PI4K2B |
| ENSSSCT00000012784 | 0.374 | 0.0009494 | XRN1 |
| ENSSSCT00000002199 | 0.383 | 0.0064858 | ARHGAP5 |
| ENSSSCT00000003848 | 0.325 | 0.0011307 | DDI2 |
| ENSSSCT00000036106 | 0.445 | 0.0035728 | ACSL4 |
| ENSSSCT00000009406 | 0.205 | 0.0005001 | APOB |
| ENSSSCT00000023932 | 0.178 | 6.70E-05 | APOB |
| ENSSSCT00000033419 | 0.639 | 0.003067 | ARNT |
| ENSSSCT00000034520 | 0.405 | 0.008657 | THBS1 |
| ENSSSCT00000027524 | 0.388 | 0.0036905 | ZEB2 |
| ENSSSCT00000004849 | 0.266 | 0.0005085 | FOXO3 |
| ENSSSCT00000007402 | 0.446 | 0.0050135 | TRIM33 |
| ENSSSCT00000015972 | 0.33 | 5.63E-06 | DNHD1 |
| ENSSSCT00000015658 | 0.433 | 0.0084566 | KDM3B |
| ENSSSCT00000012690 | 0.409 | 0.0066364 | NR2C2 |
| ENSSSCT00000002805 | 0.448 | 0.0052061 | RCOR1 |
| ENSSSCT00000005723 | 0.326 | 0.0041641 | MPDZ |
| ENSSSCT00000007987 | 0.494 | 0.0015457 | CEP250 |
| ENSSSCT00000016935 | 0.448 | 0.007483 | GLUL |
| ENSSSCT00000027703 | 0.444 | 0.0062719 | SLC2A2 |
| ENSSSCT00000033869 | 0.661 | 0.0018118 | CIITA |
| ENSSSCT00000010671 | 0.42 | 0.0048283 | GOLGA3 |
| ENSSSCT00000031275 | 0.605 | 0.0086974 | NFASC |
| ENSSSCT00000010151 | 0.478 | 0.0083181 | IFT88 |
| ENSSSCT00000003773 | 0.346 | 0.0016864 | KIF1B |
| ENSSSCT00000009139 | 0.458 | 0.001977 | BMP10 |
| ENSSSCT00000024294 | 0.407 | 0.0022376 | DIO2 |
| ENSSSCT00000016938 | 0.617 | 0.0024307 | RGSL1 |
| ENSSSCT00000000555 | 0.374 | 0.0003694 | TRHDE |
| ENSSSCT00000004941 | 0.408 | 0.008736 | PHIP |
| ENSSSCT00000019587 | 0.533 | 0.000163 | GLP2R |
| ENSSSCT00000018965 | 0.308 | 0.0028061 | ACL |
| ENSSSCT00000011114 | 0.349 | 0.0099303 | Nid1 |
| ENSSSCT00000015525 | 0.38 | 0.0068903 | APC |
| ENSSSCT00000029020 | 0.366 | 0.0003142 | PARP4 |
| ENSSSCT00000010422 | 0.351 | 0.0004177 | Mbnl2 |
| ENSSSCT00000025088 | 0.452 | 0.0061095 | AP4E1 |
| ENSSSCT00000036673 | 0.418 | 0.003863 | TNS1 |
| ENSSSCT00000024203 | 0.285 | 0.0001062 | TNS1 |
| ENSSSCT00000011303 | 0.301 | 2.96E-06 | KCNMA1 |
| ENSSSCT00000029625 | 0.457 | 0.0086656 | USP9Y |
| ENSSSCT00000030865 | 0.541 | 0.0015742 | GNPTAB |
| ENSSSCT00000008903 | 0.394 | 0.0087515 | SNRNP200 |
| ENSSSCT00000016481 | 0.42 | 0.0004511 | CBL |
| ENSSSCT00000033787 | 0.45 | 0.0012245 | LYST |
| ENSSSCT00000032717 | 0.399 | 0.0056432 | TNFSF10 |
| ENSSSCT00000007577 | 0.546 | 0.0023501 | GBP7 |
| ENSSSCT00000007727 | 0.387 | 0.0020079 | PLCB1 |
| ENSSSCT00000000153 | 0.478 | 0.0087589 | MYH11 |
| ENSSSCT00000018860 | 0.535 | 0.0055645 | LRRC37A2 |
| ENSSSCT00000030086 | 0.416 | 0.0055696 | MAP3K2 |
| ENSSSCT00000013634 | 0.529 | 0.0034302 | KLHL4 |
| ENSSSCT00000013018 | 0.44 | 0.0012294 | ADPRH |
| ENSSSCT00000004953 | 0.649 | 0.0014744 | COL12A1 |
| ENSSSCT00000016945 | 0.423 | 0.0047538 | LAMC1 |
| ENSSSCT00000006866 | 0.377 | 0.0040071 | PCMTD1 |
| ENSSSCT00000026101 | 0.588 | 0.0044189 | ZBTB34 |
| ENSSSCT00000012789 | 0.39 | 0.0026918 | TRPC1 |
| ENSSSCT00000000001 | 0.524 | 0.0091974 | CELSR1 |
| ENSSSCT00000012576 | 0.433 | 0.0056978 | ATXN7 |
| ENSSSCT00000023715 | 0.459 | 0.0065591 | SYNM |
| ENSSSCT00000006580 | 0.464 | 0.0095624 | COL14A1 |
| ENSSSCT00000010028 | 0.292 | 0.0007984 | TET2 |
| ENSSSCT00000004051 | 0.396 | 0.0030078 | FLJ42354 |
| ENSSSCT00000018477 | 0.357 | 0.0011912 | BDP1 |
| ENSSSCT00000035221 | 0.416 | 0.0016878 | OGT |
| ENSSSCT00000033775 | 0.411 | 0.0004348 | OGT |
| ENSSSCT00000034854 | 0.499 | 0.0006422 | OGT |
| ENSSSCT00000033519 | 0.465 | 0.0062346 | OGT |
| ENSSSCT00000035290 | 0.4 | 0.0029038 | OGT |
| ENSSSCT00000017321 | 0.471 | 0.0078715 | TTC21B |
| ENSSSCT00000007349 | 0.402 | 0.0042611 | NOTCH2 |
| ENSSSCT00000008162 | 0.405 | 0.0059821 | NCOA3 |
| ENSSSCT00000031900 | 0.414 | 0.0092653 | NAV2 |
| ENSSSCT00000013641 | 0.39 | 0.0066849 | DIAPH2 |
| ENSSSCT00000022643 | 0.427 | 0.0046937 | CHD1 |
| ENSSSCT00000005143 | 0.325 | 0.0002734 | FBN1 |
| ENSSSCT00000006836 | 0.314 | 0.0002321 | CYP7A1 |
| ENSSSCT00000007666 | 0.4 | 0.0022365 | FAT1 |
| ENSSSCT00000028335 | 0.507 | 0.0051335 | DIAPH2 |
| ENSSSCT00000004023 | 0.434 | 0.006854 | ZMYM4 |
| ENSSSCT00000027531 | 0.473 | 0.0037362 | PRDM2 |
| ENSSSCT00000004585 | 0.468 | 0.0086752 | NHSL1 |
| ENSSSCT00000000598 | 0.397 | 0.009481 | ITPR2 |
| ENSSSCT00000017355 | 0.41 | 0.0094685 | UBR3 |
| ENSSSCT00000030602 | 0.34 | 0.0005026 | ZNFX1 |
| ENSSSCT00000028838 | 0.427 | 0.0048934 | ELK4 |
| ENSSSCT00000015949 | 0.644 | 0.0026355 | SYT9 |
| ENSSSCT00000032267 | 0.293 | 0.0002007 | UBR4 |
| ENSSSCT00000012118 | 0.41 | 0.0023857 | ARHGAP21 |
| ENSSSCT00000028075 | 0.421 | 0.0051264 | ARHGAP21 |
| ENSSSCT00000008869 | 0.404 | 0.0038149 | ZC3H6 |
| ENSSSCT00000023548 | 0.301 | 0.000208 | UBR4 |
| ENSSSCT00000000775 | 0.433 | 0.0010783 | VWF |
| ENSSSCT00000024492 | 0.504 | 0.0019376 | ATL3 |
| ENSSSCT00000032991 | 0.39 | 0.0030135 | IL1R1 |
| ENSSSCT00000008022 | 0.402 | 0.0018341 | RBL1 |
| ENSSSCT00000011342 | 0.462 | 0.0093812 | BMPR1A |
| ENSSSCT00000010222 | 0.464 | 0.0022614 | USPL1 |
| ENSSSCT00000026288 | 0.406 | 0.0026831 | SMG7 |
| ENSSSCT00000011691 | 0.402 | 0.001724 | SEC23IP |
| ENSSSCT00000005158 | 0.336 | 9.11E-05 | GATM |
| ENSSSCT00000024092 | 0.413 | 0.0042536 | CLASP1 |
| ENSSSCT00000016679 | 0.381 | 0.0029627 | CLDN12 |
| ENSSSCT00000032012 | 0.444 | 0.007697 | CDK17 |
| ENSSSCT00000027648 | 0.451 | 0.0060652 | NAV2 |
| ENSSSCT00000000110 | 0.379 | 0.0054138 | DDX17 |
| ENSSSCT00000035135 | 0.489 | 0.0021099 | SFRP1 |
| ENSSSCT00000018209 | 0.019 | 8.66E-15 | IGFBP1 |
| ENSSSCT00000004026 | 0.413 | 0.002113 | EIF2C1 |
| ENSSSCT00000000878 | 0.339 | 0.0021036 | SLC38A2 |
| ENSSSCT00000001727 | 0.343 | 0.0002461 | FKBP5 |
| ENSSSCT00000010812 | 0.45 | 0.005437 | MED13L |
| ENSSSCT00000016977 | 0.41 | 0.0085459 | PROX1 |
| ENSSSCT00000035603 | 0.501 | 0.0080552 | DPP8 |
| ENSSSCT00000004455 | 0.422 | 0.0045923 | QKI |
| ENSSSCT00000017294 | 0.395 | 0.0034245 | BAZ2B |
| ENSSSCT00000024565 | 0.401 | 0.0050703 | BAZ2B |
| ENSSSCT00000013432 | 0.621 | 0.0066543 | ZNF81 |
| ENSSSCT00000034334 | 0.489 | 0.0011232 | TAB3 |
| ENSSSCT00000023702 | 0.281 | 0.0003279 | SELI |
| ENSSSCT00000007414 | 0.446 | 0.0020753 | LRIG2 |
| ENSSSCT00000004269 | 0.421 | 0.0058067 | ZYG11B |
| ENSSSCT00000019259 | 0.377 | 0.0062763 | ACACA |
| ENSSSCT00000008841 | 0.357 | 0.001008 | IARS |
| ENSSSCT00000029079 | 0.431 | 0.0057155 | DMD |
| ENSSSCT00000010279 | 0.37 | 0.0007987 | ALG11 |
| ENSSSCT00000016612 | 0.386 | 0.0031243 | PRDM10 |
| ENSSSCT00000024941 | 0.389 | 0.0016649 | IARS |
| ENSSSCT00000023446 | 0.457 | 0.0063806 | AKAP |
| ENSSSCT00000025176 | 0.406 | 0.0029995 | FAM73A |
| ENSSSCT00000004172 | 0.445 | 0.0071101 | FAM73A |
| ENSSSCT00000011105 | 0.461 | 0.0079215 | MTR |
| ENSSSCT00000012067 | 0.478 | 0.004598 | ZEB1 |
| ENSSSCT00000003981 | 0.381 | 0.0045206 | EPB41 |
| ENSSSCT00000019133 | 0.452 | 0.0017974 | LRRC37B |
| ENSSSCT00000004725 | 0.466 | 0.0092739 | FAM135A |
| ENSSSCT00000024938 | 0.441 | 0.0040549 | PPM1L |
| ENSSSCT00000004032 | 0.422 | 0.0082376 | Thrap3 |
| ENSSSCT00000000491 | 0.485 | 0.0018391 | LRIG3 |
| ENSSSCT00000016826 | 0.619 | 0.0024194 | NRCAM |
| ENSSSCT00000033277 | 0.439 | 0.0048687 | TSPYL2 |
| ENSSSCT00000002057 | 0.453 | 0.0068674 | ABHD2 |
| ENSSSCT00000019260 | 0.474 | 0.0031164 | ACACA |
| ENSSSCT00000006917 | 0.63 | 0.0045788 | POU2F1 |
| ENSSSCT00000017201 | 0.414 | 0.0023372 | SORBS2 |
| ENSSSCT00000032720 | 0.476 | 0.0079434 | XIAP |
| ENSSSCT00000029211 | 0.377 | 0.0036559 | XIAP |
| ENSSSCT00000017452 | 0.398 | 0.0014406 | ITGAV |
| ENSSSCT00000016956 | 0.317 | 0.0015184 | EDEM3 |
| ENSSSCT00000000614 | 0.394 | 0.0010939 | ABCC9 |
| ENSSSCT00000031569 | 0.502 | 0.0058376 | PER2 |
| ENSSSCT00000010895 | 0.414 | 0.0083987 | SSH1 |
| ENSSSCT00000032126 | 0.287 | 0.000613 | EPM2AIP1 |
| ENSSSCT00000029943 | 0.454 | 0.0091814 | EIF4B |
| ENSSSCT00000000163 | 0.457 | 0.0083801 | TIMP-3 |
| ENSSSCT00000031166 | 0.331 | 0.0042517 | TRANK1 |
| ENSSSCT00000017207 | 0.451 | 0.0087334 | TLR3 |
| ENSSSCT00000029966 | 0.362 | 0.0001204 | NOTCH3 |
| ENSSSCT00000011531 | 0.4 | 0.0028975 | ENTPD7 |
| ENSSSCT00000000994 | 0.248 | 0.0002467 | SOCS2 |
| ENSSSCT00000032138 | 0.568 | 0.0035024 | TNC |
| ENSSSCT00000016495 | 0.399 | 0.0015589 | ARHGEF12 |
| ENSSSCT00000003189 | 0.134 | 4.10E-06 | HAMP |
| ENSSSCT00000034173 | 0.202 | 0.0005862 | HAMP |
| ENSSSCT00000029960 | 0.307 | 0.0030027 | FASN |
| ENSSSCT00000004108 | 0.443 | 0.0077094 | LAMA3 |
| ENSSSCT00000025473 | 0.441 | 0.005544 | CDK5RAP2 |
| ENSSSCT00000035157 | 0.363 | 0.0011254 | SLC39A14 |
| ENSSSCT00000015473 | 0.649 | 0.0062826 | MCTP1 |
| ENSSSCT00000027129 | 0.523 | 0.0077676 | PCDHGB6 |
| ENSSSCT00000009435 | 0.403 | 0.0015883 | LPIN1 |
| ENSSSCT00000009301 | 0.442 | 0.0058489 | STRN |
| ENSSSCT00000025861 | 0.621 | 0.0044707 | SRGAP1 |
| ENSSSCT00000016687 | 0.448 | 0.0088253 | ANKIB1 |
| ENSSSCT00000023340 | 0.462 | 0.0075549 | TOPBP1 |
| ENSSSCT00000022681 | 0.6 | 0.0012277 | ZNF570 |
| ENSSSCT00000012225 | 0.493 | 0.0022203 | DIP2C |
| ENSSSCT00000022673 | 0.499 | 0.0003362 | APC2 |
| ENSSSCT00000017549 | 0.39 | 0.0003092 | NBEAL1 |
| ENSSSCT00000017139 | 0.528 | 0.0038739 | SCTR |
| ENSSSCT00000002524 | 0.372 | 0.002248 | CHD2 |
| ENSSSCT00000025402 | 0.393 | 0.0050585 | CHD2 |
| ENSSSCT00000006954 | 0.374 | 0.004147 | ATF6 |
| ENSSSCT00000026092 | 0.37 | 0.0016176 | FNDC3A |
| ENSSSCT00000003816 | 0.378 | 0.0007384 | VPS13D |
| ENSSSCT00000029631 | 0.468 | 0.0002763 | CEP170 |
| ENSSSCT00000006130 | 0.588 | 0.0063922 | ZBTB26 |
| ENSSSCT00000017851 | 0.34 | 0.0004523 | RIF1 |
| ENSSSCT00000002094 | 0.35 | 0.0006708 | SGK269 |
| ENSSSCT00000017456 | 0.398 | 0.0044834 | CALCRL |
| ENSSSCT00000012988 | 0.456 | 0.0073425 | PARP14 |
| ENSSSCT00000032523 | 0.403 | 0.0082717 | ABCA6 |
| ENSSSCT00000030093 | 0.448 | 0.0092039 | ABCA6 |
| ENSSSCT00000014817 | 0.409 | 0.0020402 | INSR |
| ENSSSCT00000031470 | 0.368 | 0.0039538 | ABCA6 |
| ENSSSCT00000031396 | 0.397 | 0.003037 | UGGT1 |
| ENSSSCT00000017266 | 0.346 | 0.0009405 | UGGT1 |
| ENSSSCT00000003787 | 0.348 | 0.0022545 | ANGPTL7 |
| ENSSSCT00000012423 | 0.382 | 0.0039702 | PLXNB1 |
| ENSSSCT00000028051 | 0.291 | 0.0004939 | ATM |
| ENSSSCT00000026078 | 0.422 | 0.0085099 | SPTBN1 |
| ENSSSCT00000016805 | 0.39 | 0.0025283 | RELN |
| ENSSSCT00000034631 | 0.478 | 0.0054452 | ATM |
| ENSSSCT00000026567 | 0.445 | 0.0065236 | C13H20ORF12 |
| ENSSSCT00000028021 | 0.527 | 0.0015582 | UBR5 |
| ENSSSCT00000013689 | 0.591 | 0.0036321 | BHLHB9 |
| ENSSSCT00000002062 | 0.473 | 0.0080381 | KIF7 |
| ENSSSCT00000031916 | 0.451 | 0.0067426 | RIN2 |
| ENSSSCT00000014494 | 0.438 | 0.0071427 | CRY2 |
| ENSSSCT00000034004 | 0.509 | 0.0011543 | Gpm6b |
| ENSSSCT00000034606 | 0.471 | 0.0005379 | Gpm6b |
| ENSSSCT00000016374 | 0.379 | 0.0015845 | EXPH5 |
| ENSSSCT00000012568 | 0.386 | 0.0009466 | PTPRG |
| ENSSSCT00000024535 | 0.408 | 0.0039842 | PIK3C2A |
| ENSSSCT00000019177 | 0.519 | 0.006079 | DGKE |
| ENSSSCT00000007807 | 0.413 | 0.0014078 | NINL |
| ENSSSCT00000014637 | 0.56 | 0.0083991 | TEF1 |
| ENSSSCT00000011926 | 0.508 | 0.0046944 | DENND1B |
| ENSSSCT00000032646 | 0.426 | 0.0087922 | CDH2 |
| ENSSSCT00000036486 | 0.469 | 0.0095303 | CDH2 |
| ENSSSCT00000001574 | 0.597 | 0.0031423 | TN-X |
| ENSSSCT00000035522 | 0.397 | 0.0015177 | STAT1 |
| ENSSSCT00000024075 | 0.402 | 0.001464 | STAT1 |
| ENSSSCT00000029213 | 0.655 | 0.0031133 | RELN |
| ENSSSCT00000007965 | 0.449 | 0.0049824 | ITCH |
| ENSSSCT00000034014 | 0.396 | 0.0010968 | PDGFRA |
| ENSSSCT00000026389 | 0.339 | 9.39E-05 | envelope glycoprotein-like |
| ENSSSCT00000009314 | 0.572 | 0.0027088 | hypothetical protein LOC100447881 |
| ENSSSCT00000023762 | 0.42 | 0.0042312 | hypothetical LOC100056402 |
| ENSSSCT00000015550 | 0.427 | 0.0076973 | protein-lysine 6-oxidase-like |
| ENSSSCT00000033273 | 0.558 | 0.0044334 | hypothetical protein LOC100154057 |
| ENSSSCT00000026108 | 0.599 | 0.0094219 | hypothetical protein LOC100515936 |
| ENSSSCT00000032071 | 0.314 | 0.0014834 | envelope glycoprotein-like |
| ENSSSCT00000014066 | 0.414 | 0.0084543 | hypothetical protein LOC100518411 |
| ENSSSCT00000004696 | 0.519 | 0.005126 | similar to chromosome 6 open reading frame 204 |
| ENSSSCT00000032264 | 0.645 | 0.0065701 | hypothetical protein LOC100519616 |
| ENSSSCT00000016357 | 0.562 | 0.0068903 | hypothetical protein LOC100524161 |
| ENSSSCT00000030906 | 0.367 | 0.0005129 | similar to K06A9.1b |
| ENSSSCT00000005737 | 0.321 | 5.57E-05 | similar to K06A9.1b |
| ENSSSCT00000030447 | 0.323 | 0.0010648 | hypothetical LOC100071238 |
| ENSSSCT00000008014 | 0.391 | 0.0001011 | hypothetical LOC458222 |
| ENSSSCT00000013031 | 0.429 | 2.93E-05 | pro-Pol polyprotein-like |
| ENSSSCT00000016402 | 0.427 | 0.0027088 | dixin-like |
| ENSSSCT00000008541 | 0.655 | 0.0066122 | pro-Pol polyprotein-like |
| ENSSSCT00000034414 | 0.435 | 0.0021327 | hypothetical protein LOC100483676 |
| ENSSSCT00000024448 | 0.248 | 9.48E-08 | envelope glycoprotein-like |
| ENSSSCT00000028150 | 0.481 | 0.003358 | interferon-induced, double-stranded RNA-activated protein kinase-like |
| ENSSSCT00000015968 | 0.284 | 7.48E-05 | similar to very large inducible GTPase 1 |
| ENSSSCT00000010606 | 0.454 | 0.0076449 | GTPase SLIP-GC-like |
| ENSSSCT00000001185 | 0.434 | 0.0098772 | phosphatidylinositol-glycan-specific phospholipase D-like |
| ENSSSCT00000033689 | 0.319 | 0.0002553 | pro-Pol polyprotein-like |
| ENSSSCT00000030032 | 0.43 | 0.0047967 | similar to leucine-rich repeat domain-containing protein |
| ENSSSCT00000022984 | 0.429 | 0.004389 | similar to Probable ubiquitin carboxyl-terminal hydrolase FAF-X |
| ENSSSCT00000033589 | 0.412 | 0.0083983 | hypothetical protein LOC100452916 |
